# eIF6 rebinding dynamically couples ribosome maturation and translation

**DOI:** 10.1038/s41467-022-29214-7

**Published:** 2022-03-23

**Authors:** Pekka Jaako, Alexandre Faille, Shengjiang Tan, Chi C. Wong, Norberto Escudero-Urquijo, Pablo Castro-Hartmann, Penny Wright, Christine Hilcenko, David J. Adams, Alan J. Warren

**Affiliations:** 1 Cambridge Institute for Medical Research, Cambridge Biomedical Campus, Keith Peters Building, Hills Rd, Cambridge, CB2 0XY UK; 2 Wellcome Trust–Medical Research Council Stem Cell Institute, Jeffrey Cheah Biomedical Centre, Puddicombe Way, Cambridge Biomedical Campus, Cambridge, CB2 0AW UK; 3 Department of Haematology, University of Cambridge School of Clinical Medicine, Jeffrey Cheah Biomedical Centre, Puddicombe Way, Cambridge Biomedical Campus, Cambridge, CB2 0AW UK; 4 Sahlgrenska Center for Cancer Research, Department of Microbiology and Immunology, Institute of Biomedicine, University of Gothenburg, 413 90 Gothenburg, Sweden; 5 Department of Pathology, Cambridge University Hospitals, Hills Road, Cambridge, CB2 0QQ UK; 6 Experimental Cancer Genetics, Wellcome Trust Sanger Institute, Wellcome Genome Campus, Hinxton, Cambridgeshire CB10 1SA UK

**Keywords:** Ribosome, Haematological diseases

## Abstract

Protein synthesis is a cyclical process consisting of translation initiation, elongation, termination and ribosome recycling. The release factors SBDS and EFL1—both mutated in the leukemia predisposition disorder Shwachman-Diamond syndrome — license entry of nascent 60S ribosomal subunits into active translation by evicting the anti-association factor eIF6 from the 60S intersubunit face. We find that in mammalian cells, eIF6 holds all free cytoplasmic 60S subunits in a translationally inactive state and that SBDS and EFL1 are the minimal components required to recycle these 60S subunits back into additional rounds of translation by evicting eIF6. Increasing the dose of eIF6 in mice in vivo impairs terminal erythropoiesis by sequestering post-termination 60S subunits in the cytoplasm, disrupting subunit joining and attenuating global protein synthesis. These data reveal that ribosome maturation and recycling are dynamically coupled by a mechanism that is disrupted in an inherited leukemia predisposition disorder.

## Abstract (web summary)

Jaako et al. discover a conserved tier of translational control that dynamically couples ribosome assembly and recycling. This mechanism is corrupted in an inherited bone marrow failure disorder associated with an increased risk of blood cancer.

## Introduction

Every minute, a growing HeLa cell synthesizes around 7500 ribosomal subunits, which decode messenger RNA to make protein through the four successive steps of translation: initiation, elongation, termination and recycling. Removal of the highly conserved nucleolar shuttling factor eukaryotic initiation factor 6 (eIF6) from the intersubunit face of the nascent large 60S ribosomal subunit is essential to license its entry into translation^1^, because eIF6 sterically inhibits the large 60S ribosomal subunit from joining to the small 40S subunit to form an actively translating ribosome^2,3^. eIF6 is initially recruited to pre-60S ribosomal subunits in the nucleolus^4^. Following export of the pre-60S particles to the cytoplasm, the GTPase EFL1 (elongation factor-like 1) and its cofactor SBDS (Shwachman-Bodian-Diamond syndrome) evict eIF6 during the final step in maturation of the nascent 60S subunit^5–11^.

Disruptive variants in both SBDS^12^ and EFL1^13^ cause the inherited leukemia predisposition disorder Shwachman-Diamond syndrome (SDS)^14^. Missense variants in eIF6 can bypass the fitness defect of yeast cells lacking the SBDS orthologue Sdo1 by reducing eIF6 binding to the 60S subunit^7^. In addition, diverse somatic genetic events including point mutations, interstitial deletion, and reciprocal chromosomal translocation rescue the germline ribosome defect in SBDS-deficient hematopoietic cells either by reducing eIF6 expression or by disrupting the interaction of eIF6 with the 60S subunit^15,16^. The observation that mutations in eIF6 can rescue the defects in ribosomal subunit joining and translation initiation observed in SBDS-deficient cells^15^ raises the possibility that SBDS and EFL1 may have a more general role in translation beyond their function in nascent 60S subunit maturation. Cryo-electron microscopy (cryo-EM) studies further support this hypothesis by revealing that eIF6 is bound to 60S ribosome quality control intermediates^17,18^, suggesting that there are some contexts in which eIF6 may rebind to mature 60S ribosomal subunits in vivo.

In eukaryotes, translation termination begins with the recognition of a stop codon in the A site of the 80S ribosome by the release factors eRF1 and GTP-bound eRF3^19^. Peptide release is temporally coupled to splitting of the 80S ribosome into a free 60S and a 40S subunit bound to deacylated tRNA and mRNA by the essential ATP-binding cassette protein Rli1 (yeast)/ABCE1 (mammals)^20,21^. The deacylated tRNA is subsequently removed, promoting dissociation of the 40S subunit from the mRNA^20,22^. ABCE1 blocks 40S rebinding to the 60S subunit by sterically hindering the formation of an intersubunit bridge between the 60S protein uL14^23^ and the 40S rRNA helix h44^24^. However, the possibility that eIF6 rebinding might similarly sequester post-termination recycled 60S subunits in a translationally inactive state has not been addressed. Dissociated 40S and 60S subunits may immediately re-engage in further rounds of translation initiation or alternatively, in conditions of stress, enter a reservoir of translationally inactive 80S ribosomes^25–28^, that can again be recycled in an ABCE1-dependent manner^29^. Interestingly, ribosome recycling becomes critical for ribosome homeostasis during erythroid differentiation, as the natural loss of ABCE1 limits ribosome availability and results in the accumulation of post-termination, unrecycled ribosomes in the 3′UTRs of mRNAs^30^.

Here, we test the hypothesis SBDS and EFL1 act as general eIF6 release factors to regulate post-termination 60S ribosomal subunit recycling. Using cryo-EM, we show that eIF6 binds to the majority of free cytoplasmic 60S subunits in mammals, thereby holding them in a translationally inactive state. We reveal that SBDS and EFL1 are the minimal components required to evict 60S-rebound eIF6 and recycle post-termination 60S subunits back into the actively translating pool. Consistent with the requirement for efficient ribosome recycling during erythropoiesis, graded overexpression of eIF6 in mice perturbs late steps in erythroid differentiation by sequestering free 60S subunits, blocking subunit joining and attenuating global translation. Our data support a wider role for SBDS and EFL1 as translational regulators that dynamically couple 60S subunit maturation with ribosome recycling through the release of rebound eIF6.

## Results

### eIF6 holds free cytoplasmic 60S subunits in a translationally inactive state in vivo

**Fig. 1:**
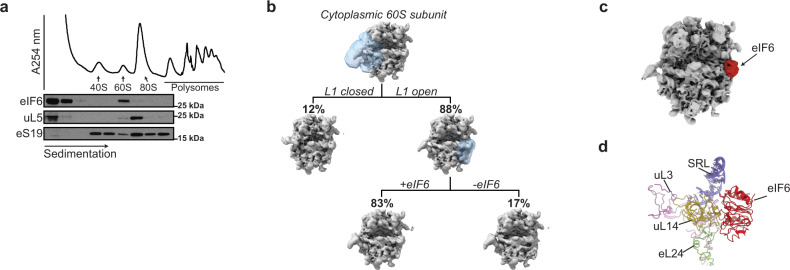
eIF6 maintains free mammalian 60S subunits in a translationally inactive state. **a** Sucrose gradient sedimentation of eIF6 in cell extracts prepared from murine c-kit+ bone marrow cells. The distribution of eIF6, uL5, and eS19 was visualized by immunoblotting. Data are representative of at least five independent experiments. **b** Cryo-EM classification scheme to quantify the frequency of eIF6-bound 60S subunits in the cytoplasm. See “Methods” for further details. **c** eIF6 binds the intersubunit face of free cytoplasmic 60S subunits. Crown views of the cryo-EM maps of native 60S–eIF6 complexes isolated from murine c-kit+ bone marrow cells. eIF6 is highlighted by the red color. **d** Atomic model for the murine 60S ribosomal subunit bound to human eIF6.

We set out to test the hypothesis that eIF6 maintains free cytoplasmic 60S subunits in a translationally inactive state in primary hematopoietic cells in vivo. Immunoblotting of cell extracts purified from primary murine c-kit+ bone marrow cells revealed that around 14% of the eIF6 protein co-migrated with free 60S ribosomal subunits, while the majority was distributed in the free fraction (Fig. 1a). Single particle cryo-electron microscopy (cryo-EM) analysis of free 60S particles purified from primary murine c-kit+ bone marrow cells revealed that eIF6 is stably bound to the intersubunit face of at least 83% of cytoplasmic mature 60S subunits (Fig. 1b).

At an overall resolution of 3.1 Å, our cryo-EM reconstructions allowed us to build and refine atomic models of murine eIF6 bound to the 60S ribosomal subunit (Fig. 1c and Supplementary Fig. 1). Conserved in archaea and eukaryotes, eIF6 is a member of the pentein protein superfamily with five-fold pseudosymmetry^31^. Consistent with the previous structures from yeast^3^, *Tetrahymena*^32^ and human cells^15^, murine eIF6 sterically inhibits 40S ribosomal subunit joining by binding to a conserved site on the intersubunit face of the 60S subunit involving the C terminus of uL14, the sarcin-ricin loop (SRL), uL3 (residues 58–71) and the N terminus of eL24 (Fig. 1d). We conclude that in primary murine hematopoietic cells, eIF6 holds all free 60S ribosomal subunits in a translationally inactive state by binding to the 60S intersubunit face. These data support the hypothesis that eIF6 must be released from the 60S ribosomal subunit to allow 80S ribosome assembly^1^. However, we were unable to discriminate nascent 60S–eIF6 complexes versus eIF6 rebound to mature 60S subunits.

### Endogenous eIF6 can rebind mature cytoplasmic 60S subunits

The ribosome quality control (RQC) pathway recognizes and rescues stalled translation complexes. Following ribosome dissociation, components of the RQC complex remain bound to the 60S subunit together with eIF6^17,33^. Taken together with the finding that eIF6 is bound to virtually all mature cytoplasmic 60S ribosomal subunits, we hypothesized that during canonical translation termination (and RQC), eIF6 might rebind to mature 60S particles and require dynamic recycling by SBDS and the GTPase EFL1.

**Fig. 2:**
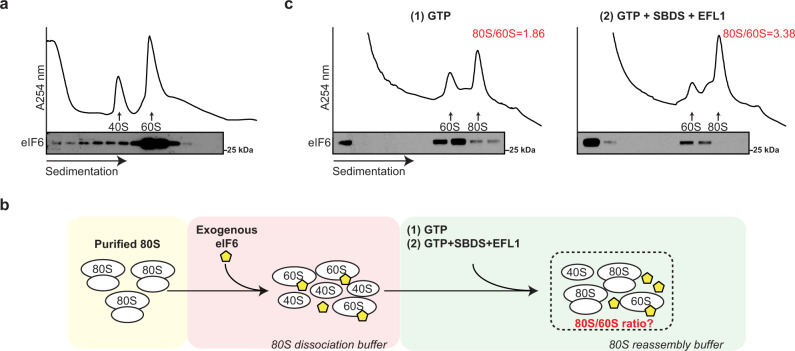
SBDS and EFL1 catalyze GTP-dependent release of rebound eIF6 from mature cytoplasmic 60S ribosomal subunits. **a** Sucrose gradient sedimentation of c-Kit+ bone marrow cell extracts (without cycloheximide) lysed in 20 mM Hepes pH 7.5, 2 mM Mg(CH_3_COO)_2_, 500 mM KCl and incubated for 10 min at 37 °C to allow eIF6 rebinding. eIF6 was detected by immunoblotting. Data are representative of two independent experiments. **b** Schematic overview of in vitro eIF6 release assay. See “Methods” section for further details. **c** Sucrose gradient sedimentation of reconstituted eIF6 release reaction mixes. Immunoblotting was used to detect eIF6. The ratio of 80S monosomes to 60S subunits is indicated. Shown is a representative experiment out of a total of two independent experiments.

To support this hypothesis, we first tested the ability of eIF6 to rebind mature 60S particles that had been dissociated from 80S couples. Using immunoblotting, we examined the distribution of endogenous eIF6 following sucrose gradient fractionation of cell extracts prepared from c-kit+ murine bone marrow cells in 80S dissociating conditions (2 mM Mg(CH_3_COO)_2_, 500 mM KCl). In contrast to non-dissociating conditions where eIF6 predominantly migrates in the free fraction (Fig. 1a), eIF6 comigrated almost entirely with the 60S subunit (Fig. 2a). Consistent with previous work^1^, we conclude that endogenous eIF6 can rebind mature cytoplasmic 60S subunits in mammalian cells.

### SBDS and EFL1 are sufficient to release eIF6 rebound to 60S subunits

We next examined whether human SBDS, EFL1, and GTP are sufficient to promote the release of eIF6 rebound to mature cytoplasmic 60S subunits. We biochemically reconstituted an ex vivo assay that coupled eIF6 release from 60S subunits to their reassembly into 80S ribosomes by adding recombinant human SBDS and EFL1 to eIF6-loaded 60S subunits isolated from c-kit+ bone marrow cells. A schematic overview of the assay is shown in Fig. 2b. As shown in the representative experiment in Fig. 2c, compared with GTP alone (left panel), the addition of SBDS, EFL1, and GTP (right panel) to eIF6-loaded 60S subunits promoted redistribution of eIF6 into the free fraction of the sucrose gradient as detected by immunoblotting, with a concomitant 1.8-fold increase in 80S ribosome reassembly. We conclude that in the presence of GTP, SBDS, and EFL1 are sufficient to release eIF6 that has rebound to mature 60S particles. These data provide biochemical support for the hypothesis that SBDS and EFL1 function as general release factors with dual roles in nascent 60S subunit maturation and in ribosome recycling.

### Genetic interactions between SBDS, EFL1, and eIF6

We reasoned that if eIF6 dynamically rebinds to post-termination 60S ribosomal subunits, increasing the dose of eIF6 in vivo would titrate out free 60S subunits to impair ribosomal subunit joining, reduce global protein synthesis and induce growth defect. Consistent with this hypothesis, ubiquitous overexpression of wild type eIF6 (but not eIF6 missense mutants identified in SDS hematopoietic cells that map to the interface with the 60S subunit) induces late larval lethality in *Drosophila*^15^. Furthermore, overexpression of SDS patient-derived eIF6 missense mutations can fully rescue the lethality of Sbds-deficient flies^15^.

**Fig. 3:**
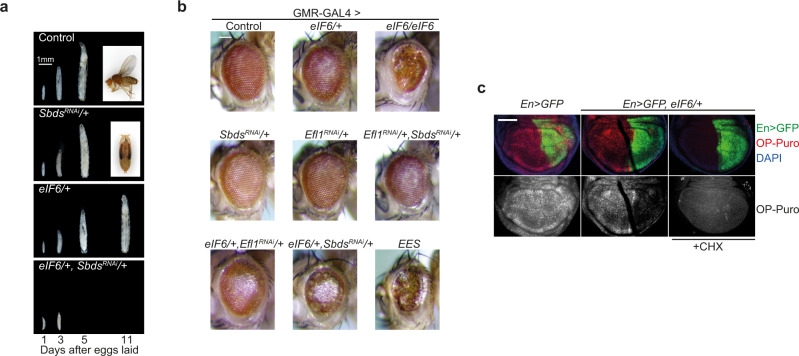
Genetic interactions between *Sbds*, *Efl1*, and *eIF6*. **a** Increased eIF6 dosage enhances the growth defects of Sbds-deficient *Drosophila*. Flies were photographed at 1, 3, 5, and 11 days after egg laid. Scale bar, 1 mm. **b** Genetic interactions between *Sbds*, *Efl1*, and *eIF6* in the *Drosophila* eye. Representative photomicrographs of adult eyes from flies with the indicated genotypes. *EES*, abbreviation of (*eIF6/+, Efl1*^*RNAi*^*/+, Sbds*^*RNAi*^*/+*). Scale bar, 100 μm. **c** Overexpression of eIF6 suppresses global protein synthesis in *Drosophila* wing disc cells. Third instar larval wing disc cells with the indicated genotypes were immunostained to reveal OP-Puro incorporation (red, gray). Posterior wing disc cells are marked with GFP; nucleus is blue (DAPI), scale bar: 100 μm. Data are representative of three independent experiments including ten wing discs each.

To further test the in vivo genetic interactions between Sbds and eIF6, we depleted *Sbds* using RNAi^15^, allowing flies to develop to adult stage albeit more slowly compared with wild type controls (Fig. 3a and Supplementary Fig. 2). At 29 °C, 5.4% of *Sbds*-depleted flies develop from pupae to adults (*n* = 269, 3 replicates); at 25 °C, 54.5% of pupae develop to adults (*n* = 666, 6 replicates). RNAi-mediated depletion of *Sbds* enhanced the growth defect induced by ubiquitous overexpression of eIF6, causing early larval lethality (Fig. 3a) due to an enhanced defect in ribosomal subunit joining as revealed by sucrose gradient sedimentation of extracts prepared from second instar larvae (Supplementary Fig. 2). Selective overexpression of eIF6 in the developing ommatidia induced a small, rough eye phenotype (Supplementary Fig. 3) that was enhanced either by doubling the dose of eIF6 or by depleting *Sbds* or *Efl1* by RNAi (Fig. 3b, Supplementary Fig. 3). Selective overexpression of eIF6 in the *Drosophila* wing disc reduced global protein synthesis as measured by O-propargyl-puromycin (OP-puro) incorporation (Fig. 3c). These genetic data support the hypothesis that SBDS and EFL1 function in mobilizing eIF6 that has rebound to cytoplasmic 60S ribosomal subunits in vivo.

### eIF6 dose-dependent inhibition of ribosomal subunit joining in vivo

**Fig. 4:**
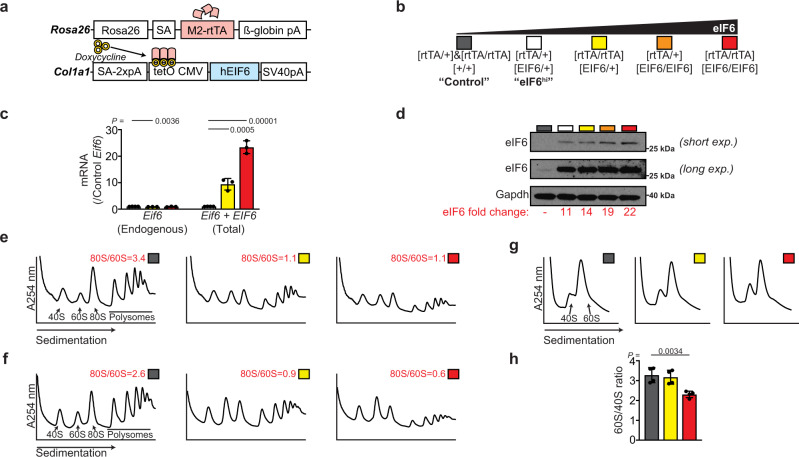
eIF6 binds post-termination 60S subunits to prevent ribosomal subunit joining. **a** Schematic overview of the transgenic Dox-inducible eIF6 overexpression system. **b** Breeding strategy for graded overexpression of eIF6, with color coding of indicated genotypes. **c** Quantitative real-time PCR of *EIF6* transcript levels (*n* = 4, 3, and 3 biologically independent samples per genotype). **d** eIF6 protein immunoblotting analysis in extracts from cultured c-Kit+ bone marrow cells derived from the indicated mouse strains after 24 h of Dox induction. **e**, **f**. Sucrose gradient sedimentation of extracts (including cycloheximide) from cultured c-Kit+ bone marrow cells derived from the indicated mouse strains. Dox induction, 24 h. Buffers in (**e**) and (**f**) contain 50 mM or 200 mM KCl, respectively. Shown is representative of two independent experiments. **g** Sucrose gradient sedimentation of extracts prepared in absence of magnesium to dissociate 80S ribosomes and polysomes. **h** Quantification of the 60S:40S subunit ratios shown in (**g**) (*n* = 3 per genotype). Student’s *t* test was used to determine statistical significance. Two-tailed *P* values are shown. All graphs show mean ± standard deviation.

We set out to further validate the hypothesis that eIF6 dynamically rebinds to post-termination cytoplasmic 60S ribosomal subunits by engineering a transgenic eIF6 mouse strain that permits doxycycline (Dox, tetracycline analogue)-inducible and graded overexpression of the human *EIF6* transgene by constitutively expressing the M2-reverse tetracycline transactivator (M2-rtTA) at the *Rosa26* promoter^34^ (Fig. 4a, b). M2-rtTA is a mutant of rtTA that has increased stability, reduced background expression and improved inducibility in the presence of Dox^35^. This transgenic mouse strain exhibits widespread constitutive expression of M2-rtTA, allowing for Dox-inducible transactivation of the human *EIF6* cDNA. Mice carrying one or two copies of the *M2-rtTA* transgene but no *EIF6* transgene were used as controls to exclude toxicity or confounding effects of doxycycline alone. We adjusted the level of eIF6 overexpression by breeding animals that were heterozygous or homozygous for the *M2-rtTA* at the *Rosa26* locus and carried one or two copies of the human *EIF6* transgene at the *Col1a1* locus (Fig. 4b). To evaluate the level of *EIF6* transgene expression, we treated cultured c-Kit+ bone marrow cells with Dox and performed quantitative real-time PCR to measure *EIF6* mRNA. We designed two sets of primers to distinguish endogenous mouse *Eif6* mRNA from total (endogenous + transgene) *EIF6* mRNA to verify the transgene copy number-dependent increase in total *EIF6* expression (a 9.4-fold and 23.4-fold increase in [M2-rtTA/M2-rtTA; EIF6/+] and [M2-rtTA/M2-rtTA; EIF6/EIF6] cells, respectively; Fig. 4c). The increase in *EIF6* mRNA led to an increased abundance of eIF6 protein (range: 11- to 22-fold; Fig. 4d).

Next, we assessed the impact of increasing doses of eIF6 on ribosome assembly in vivo by fractionating cell extracts in the presence of cycloheximide from Dox-treated c-Kit+ bone marrow cells by sucrose gradient sedimentation. An increased dose of eIF6 promoted a reduction in the 80S:60S ratio, consistent with a subunit-joining defect (Fig. 4e). Parallel experiments using high salt buffer to specifically dissociate inactive mRNA-free 80S  monosomes^36^, further highlighted the eIF6 dose-dependent reduction in actively translating 80S ribosomes (Fig. 4f). Finally, by using a magnesium-free buffer system, we observed that the ratio of 60S to 40S subunits was preserved with an intermediate dose of eIF6 (Fig. 4g). Although higher eIF6 overexpression resulted in a relative decrease in 60S subunits **(**Fig. 4h**)**, this is likely to be a secondary consequence of the profound reduction in global protein synthesis. We conclude that graded eIF6 overexpression induces a dose-dependent ribosomal subunit joining defect in vivo. Importantly, the observed ribosomal subunit joining defect upon eIF6 overexpression closely mimics the subunit joining defect caused by eIF6 retention on the 60S subunit that is observed in *Sbds*- or *Efl1* deficient mice or patient-derived lymphoblasts^8,9,13,37^. Taken together with our genetic data in *Drosophila*, we propose that the most logical interpretation of these findings is that eIF6 rebinds to post-termination recycling 60S subunits from which it is dynamically recycled by SBDS and EFL1. These data support the hypothesis that SBDS and EFL1 translationally activate nascent 60S subunits and in addition act as general eIF6 release factors that dynamically recycle eIF6-bound post-termination 60S subunits back into additional rounds of translation.

### Terminal erythroid differentiation is sensitive to eIF6 dosage

We reasoned that during mammalian hematopoiesis, the erythroid lineage might be particularly sensitive to an increased dose of eIF6 and aberrant ribosome homeostasis due to the increased dependence of terminal erythroid differentiation on ribosome recycling because of natural loss of the ribosome recycling factor ABCE1^30^. To test this hypothesis, we induced eIF6 overexpression in vivo in transgenic mice.

Detailed analysis of mice carrying two copies of the *M2-rtTA* transgene and either one or two copies of the *EIF6* transgene was precluded because of the rapid weight loss induced in these animals. By contrast, mice that were heterozygous for both transgenes (*M2-rtTA/+*; *EIF6/+*, herein called eIF6^hi^ mice) did not lose weight acutely in response to Dox administration **(**Supplementary Fig. 4**)**. We, therefore, restricted our analysis to eIF6^hi^ mice.

Immature (lineage-, Sca-1+, c-Kit+; LSK), myeloid (preGM/GMP) and erythroid (preCFU-E/CFU-E) hematopoietic progenitor cells isolated from Dox-treated eIF6^hi^ mice showed a 2–4 fold increase in *EIF6* mRNA (Supplementary Fig. 5a), while sucrose gradient sedimentation analysis of extracts from cultured c-Kit+ bone marrow cells showed accumulation of free 40S and 60S subunits compared with control (Supplementary Fig. 5b). Immunoblotting revealed a robust increase in eIF6 protein across the gradient (Supplementary Fig. 5c). Compared with controls, the overexpressed eIF6 protein predominantly accumulated in the cytoplasm of freshly isolated bone marrow cells in eIF6^hi^ mice (Supplementary Fig. 5d).

**Fig. 5:**
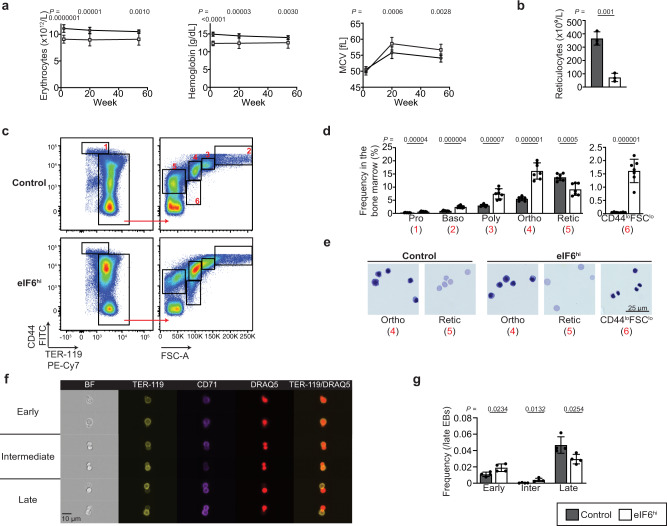
Increased eIF6 dosage impairs erythroblast enucleation in mice. **a** Increased eIF6 dosage causes macrocytic anemia. Hematological parameters including hemoglobin concentration, erythrocyte count and mean corpuscular volume (MCV) are shown over the indicated time-course of Dox induction for eIF6^hi^ mice versus control. 2 and 20 weeks, *n* = 13 and 15 animals per genotype; 1 year, *n* = 12 and 13 animals per genotype. Hemoglobin at 2 weeks, *P* = 0.00000000002. **b** Reticulocyte counts (*n* = 3 animals per genotype). **c** Representative flow cytometry analysis of erythroid precursors in control versus eIF6^hi^ bone marrow. Gated populations are designated 1–6 in red. **d** Frequency of erythroid precursors in the bone marrow (*n* = 7 biologically independent samples per genotype), corresponding to gated populations 1–6 in the flow cytometry analysis. Pro, proerythroblast; Baso, basophilic erythroblast; Poly, polychromatic erythroblast; Ortho, orthochromatic erythroblast; Retic, reticulocyte. **e** Morphology of erythroid precursors, corresponding to populations 4–6 by flow cytometry. **f** Representative images of enucleating erythroblasts, defined by Amnis ImageStream IDEAS gating strategy, shown in Supplementary Fig. 11. **g** Frequencies of enucleating erythroblasts within the late erythroblast population (corresponding to gate 6 in IDEAS gating strategy), in the bone marrow after 2 weeks of Dox administration (*n* = 4 biologically independent samples per genotype). All graphs show mean ± standard deviation. Student’s *t* test was used to determine statistical significance. Two-tailed *P* values are shown.

After two weeks of Dox administration, eIF6^hi^ mice developed persistent macrocytic anemia with a significant reduction in the reticulocyte count compared with controls (Fig. 5a, b). While the platelet count increased, the total white blood cell count was unaffected (Supplementary Fig. 6). Histological examination of the bone marrow revealed erythroid hyperplasia in eIF6^hi^ mice, with an increased frequency of erythroid precursors compared with controls (Supplementary Fig. 7a). In addition, the spleen was enlarged in eIF6^hi^ mice (Supplementary Fig. 7b), due to marked expansion of erythroid precursors (Supplementary Fig. 7c).

To further characterize hematopoiesis in the eIF6^hi^ mice, we analyzed bone marrow cells by flow cytometry^38,39^, using the gating strategy shown schematically in Supplementary Fig. 8. The eIF6^hi^ mice showed no significant differences in overall bone marrow cellularity relative to controls (Supplementary Fig. 9a). Although the frequency of myeloid and multipotent progenitors (preGM and MPPs) and granulocyte precursors^40^ decreased (Supplementary Figs. 9b, 10), the frequency of erythroid progenitors (preCFU-E and CFU-E) (Supplementary Fig. 9b) and precursor cells (Fig. 5c–e) was significantly increased. A similar increase in the frequency of erythroid precursors was detected by flow cytometry in the spleen (Supplementary Fig. 9c). Within the bone marrow, we identified an abnormal population of orthochromatic erythroblast-like cells (CD44^lo^ FSC^lo^) containing a highly condensed nucleus and low cytoplasmic volume (Fig. 5c–e).

We hypothesized that an increased dose of eIF6 might impair erythroblast enucleation during the terminal steps of erythroid differentiation, promoting the accumulation of orthochromatic erythroblast-like cells, but reducing the numbers of reticulocytes. To test this, we applied Amnis ImageStream technology^41,42^ to visualize active nuclear extrusion by bone marrow erythroblasts, dividing the process into early, intermediate and late stages (Fig. 5f and Supplementary Fig. 11). Compared with controls, in Dox-treated eIF6^hi^ mice we classified more erythroblasts in the early or intermediate stages of enucleation compared with late steps (Fig. 5g). We conclude that an increased dose of eIF6 impairs terminal enucleation of orthochromatic erythroblasts in vivo.

We next set out to determine whether the eIF6-dependent erythroid differentiation defect was intrinsic to eIF6^hi^ hematopoietic cells. Consistent with this hypothesis, ex vivo differentiation of CFU-Es/proerythroblasts isolated from Dox-treated eIF6^hi^ mice recapitulated the eIF6-dependent defect in terminal erythropoiesis (Supplementary Fig. 9d). Furthermore, non-competitive transplantation of bone marrow cells from eIF6^hi^ mice into lethally irradiated wild type congenic recipients also recapitulated the eIF6 dose-dependent hematopoietic abnormalities (Supplementary Fig. 12). Taken together, our data indicate that the terminal erythroid maturation defects are intrinsic to eIF6^hi^ hematopoietic cells.

### Attenuated protein synthesis impairs terminal erythroblast enucleation

**Fig. 6:**
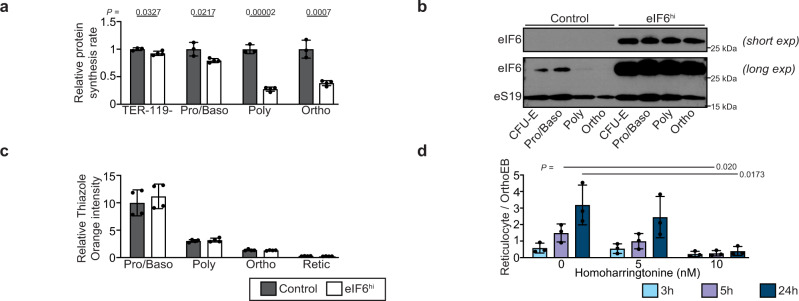
Increased eIF6 dosage impairs erythroblast enucleation by attenuating protein synthesis. **a** OP-Puro incorporation in the indicated bone marrow cells in vivo after two weeks of Dox administration (*n* = 3 and 4 biologically independent samples per genotype). Median fluorescence intensities were normalized against the respective control cell populations. **b** Expression of eIF6 in CFU-E erythroid progenitor cells and erythroid precursors in vivo after two weeks of Dox treatment. Immunoblots are shown for eIF6 and eS19 using extracts generated from identical numbers of the indicated bone marrow cells. Shown is representative of two independent experiments. CFU-E progenitor cells are defined as CD71+ TER-119- bone marrow cells. **c** Total cellular nucleic acid content in vivo during terminal erythroid differentiation. Freshly isolated bone marrow cells (*n* = 4 biologically independent samples per genotype) were stained with thiazole orange. Thiazole orange intensities are shown relative to CD44+ TER-119- non-erythroid bone marrow cells. **d** Enucleation of FACS-purified wild type orthochromatic erythroblasts in culture after 3, 5, or 24 hr treatment with homoharringtonine (*n* = 3 independent experiments). Enucleation efficiency is expressed as the ratio of reticulocytes to orthochromatic erythroblasts. All graphs show mean ± standard deviation. Student’s *t* test was used to determine statistical significance. Two-tailed *P* values are shown.

We hypothesized that increasing the dose of eIF6 would alter ribosome homeostasis during erythropoiesis by shifting the equilibrium towards ribosomal subunit dissociation, thereby attenuating protein synthesis. To test this, we quantified the rate of global protein synthesis in erythroid cells in vivo by measuring OP-puro incorporation. Indeed, compared with controls, we observed a significant decrease in OP-puro incorporation in late poly- and orthochromatic erythroid precursors from Dox-treated eIF6^hi^ mice (Fig. 6a). These data demonstrate that eIF6 overexpression impairs terminal erythroid differentiation by a mechanism that directly or indirectly attenuates protein synthesis.

We reasoned that the reduced rate of protein synthesis in late erythroblasts from Dox-treated eIF6^hi^ mice likely reflects altered ribosome homeostasis because of an increase in the relative ratio of eIF6 to ribosomes during terminal erythroid differentiation. To test this hypothesis, we sorted identical numbers of erythroid progenitor and precursor cells from Dox-treated mice and performed immunoblotting to visualize eIF6 and eS19 (as a marker for cellular ribosome levels). In control mice, the levels of eIF6 and eS19 peaked in early erythroblasts and progressively declined during terminal erythroid differentiation (Fig. 6b). By contrast, erythroblasts in Dox-induced eIF6^hi^ mice exhibited sustained high levels of eIF6 (Fig. 6b). The relative intensity of thiazole orange staining (correlating with cellular ribosomal RNA content) of freshly isolated erythroblasts was consistent with a progressive decline in cellular ribosome levels during terminal erythroid maturation (Fig. 6c). Taken together, these results indicate that an increased dose of eIF6 relative to ribosomal subunits is sustained in the eIF6^hi^ mice throughout erythropoiesis. Erythroid differentiation is likely susceptible to increased eIF6 dosage due to the combined shutdown in new ribosome synthesis in early erythroblasts^43^ together with the loss of effective ribosome recycling through natural loss of the ribosome recycling factor ABCE1 during terminal differentiation^30^. We propose that the increased dose of eIF6 titrates out recycled post-termination 60S subunits during late erythroid differentiation to push the equilibrium in favor of ribosomal subunit dissociation, impaired translation initiation and attenuated protein synthesis. Finally, consistent with the impact of eIF6 overexpression on terminal erythroid differentiation, inhibition of protein synthesis with the translational elongation inhibitor homoharringtonine in prospectively isolated wild-type orthochromatic erythroblasts recapitulated the erythroblast enucleation defect observed in eIF6^hi^ mice (Fig. 6d).

## Discussion

**Fig. 7:**
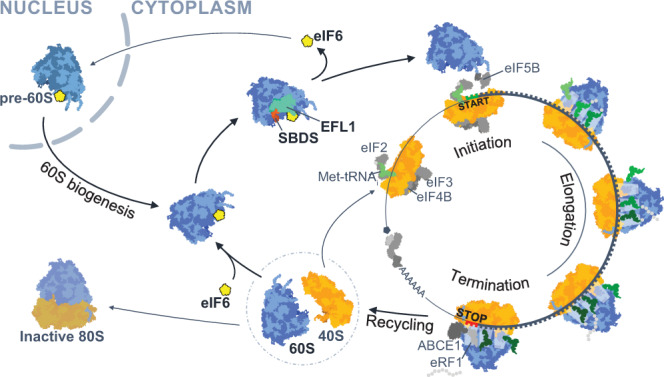
Model illustrating how dynamic rebinding of eIF6 couples ribosome maturation and translation. eIF6 functions as a ribosome anti-association factor to hold nascent pre-60S and mature post-termination 60S subunits in a translationally inactive state. SBDS and EFL1 couple nascent 60S subunit maturation and ribosome recycling by acting as general eIF6 release factors.

In this study, we have identified a critical role for the SBDS and EFL1 proteins in the regulation of translation initiation by acting as general eIF6 release factors. Using cryo-EM, we provide direct evidence that eIF6 holds virtually all free cytoplasmic 60S subunits in mammalian cells in a translationally inactive state and show that SBDS and EFL1 are the minimal components required to recycle eIF6 that has rebound to post-termination 60S subunits. Depletion of Sbds or Efl1 exacerbates the growth defects caused by eIF6 overexpression in *Drosophila* in vivo, while eIF6 overexpression in mice causes a dose-dependent defect in ribosomal subunit joining by rebinding and titrating out post-termination 60S subunits from active translation. The observation that inactive 80S monosomes accumulate in eIF6 haploinsufficient mice^44^ also supports the hypothesis that eIF6 prevents the formation of inactive 80S monosomes by binding to post-termination 60S subunits. Taken together, our data support a role for SBDS and EFL1 in regulating ribosome homeostasis by coupling the final step in cytoplasmic 60S subunit maturation with post-termination 60S ribosomal subunit recycling (Fig. 7).

Translation of mRNA occurs in four steps: initiation, elongation, termination, and ribosome recycling. During the normal translation cycle, once the ribosome reaches the stop codon of the mRNA, eRF1 and eRF3 recognize the stop codon and trigger hydrolysis of the nascent chain. Upon dissociation of eRF3, 80S ribosomes are recycled by recruitment of the ATPase ABCE1 to regenerate free 40S and 60S subunits^20^. This process maintains ribosome homeostasis by promoting additional rounds of translation initiation. Following 80S ribosome dissociation, the free 60S subunit may re-enter a new round of translation by binding a 48S pre-initiation complex to form an elongation competent 80S. Alternatively, it may bind an empty 40S subunit to form a vacant mRNA-free 80S monosome. A third possibility is that post-termination 60S subunits bind eIF6 to maintain the cytoplasmic pool of free ribosomal subunits in a translationally inactive state.

This begs the question of how translationally inactive eIF6-bound 60S subunits are recycled back into active translation. Genetically, depletion of SBDS and EFL1 reduces global protein synthesis due to the defect in ribosomal subunit joining caused by eIF6 retention on the intersubunit face of the 60S subunit^7,13,15^. While SBDS and EFL1 are known to release eIF6 during the final cytoplasmic step in nascent 60S maturation, the marked reduction in protein synthesis in SBDS and EFL1-deficient cells suggested to us that these factors may have a broader role as general release factors that liberate rebound eIF6 in a number of different contexts such as during post-termination ribosome recycling.

Although eIF6 was shown to bind to free 60S subunits by immunoblotting of mammalian cell extracts fractionated by sucrose gradient sedimentation^1^, the stoichiometry of this interaction in vivo remained unclear. In this study, we show that increasing the dose of eIF6 in vivo alters ribosome homeostasis by sequestering all free post-termination cytoplasmic 60S subunits, impairing ribosomal subunit joining and reducing 80S assembly. The subunit joining defect induced by eIF6 overexpression mimics the consequences of SBDS or EFL1 deficiency in SDS patient cells, *Dictyostelium*, mice and zebrafish^8,9,13,37^ and is exacerbated by concomitant depletion of either SBDS or EFL1. Our data, therefore, suggest that increasing the dose of eIF6 alters ribosome homeostasis by exceeding the capacity of endogenous SBDS and EFL1 to evict eIF6 from dynamically recycling 60S subunits.

Our findings suggest that the inability to dynamically upregulate recycling of post-termination ribosomes back into active translation at key time points during development may be a critical facet of SDS pathogenesis. This is exemplified by the defect in erythroid differentiation we observed in mice expressing an increased dosage of eIF6. Our model provides a more satisfactory explanation of why diverse mosaic somatic genetic events, including point mutations, interstitial deletion and reciprocal chromosomal translocation involving *EIF6* may confer a selective advantage in SBDS-deficient hematopoietic cells^15^ by disrupting the expression of eIF6 or its interaction with cytoplasmic (but not nuclear) 60S subunits, while still preserving ribosome biogenesis. Indeed, SDS-related somatic *EIF6* missense mutations that reduce eIF6 dosage or binding to cytoplasmic 60S subunits suppress the ribosome assembly and protein synthesis defects across multiple SBDS-deficient species including yeast, *Dictyostelium*, *Drosophila* and human cells^15^. Taken together, these genetic and biochemical data support a major role for SBDS and EFL1 in regulating cytoplasmic ribosome homeostasis and translational control.

As our transgenic mice overexpressing eIF6 recapitulate the defect in ribosome assembly observed in SDS, this model may provide a tool to further dissect SDS pathogenesis. Like germline depletion of Sbds or Efl1 in mice^13,45,46^, high doses of eIF6 are not systemically tolerated. However, future studies combining the *EIF6* transgene with tissue-specific tetracycline transactivator mouse strains will bypass this limitation, harnessing the full potential of this model. Finally, our inducible eIF6 transgenic mouse model may find utility in the development of therapeutic strategies to restore cytoplasmic ribosome homeostasis in SDS by modulating the rebinding of eIF6 to cytoplasmic 60S subunits.

## Methods

### Generation of transgenic eIF6 mouse strain

Gibson assembly was used to clone a full-length human *EIF6* cDNA containing Kozak sequence (5′-ATCACG-3′) into the EcoRI site of pBS31 vector, which was in turn used to target the KH2 embryonic stem (ES) cell line^34^. The engineered ES cells were injected into E3.5 C57BL/6 blastocysts to generate chimeric mice. Mice were backcrossed into the C57BL/6 background for at least three generations. PCR was used to genotype the *Rosa26* locus (5′-AAAGTCGCTCTGAGTTGTTAT-3′; 5′-GCGAAGAGTTTGTCCTCAACC-3′; 5′-GGAGCGGGAGAAATGGATATG-3′; WT product: 600 bp; Insert product: 300 bp) and the *Col1a1* locus (5′-TCCCTCACTTCTCATCCAGATATT-3′; 5′-AGTCTTGGATACTCCGTGACCATA-3′; 5′-GGACAGGATAAGTATGACATCATCAA-3′; WT product: 1092 bp; Insert product: 455 bp). The *EIF6* transgene was induced in vivo by administering Dox in the food (ssniff-Spezialdiäten GmbH; 2000 mg/kg).

Mice were maintained in specific pathogen-free conditions and all procedures were regulated under UK Home Office Animals (Scientific Procedures) Act 1986 under project license 70/8406. All experiments were performed using adult (8–12 weeks old) female and male mice with littermate controls. Mice were maintained in a standard SPF facility (12 light/12 dark cycle, 19–23 degrees Celsius with 40–60% humidity)

### Peripheral blood analysis

Peripheral blood was collected from the tail vein into Microvette® 500 K3E tubes (Sarstedt) and cellularity analyzed using a Woodley ABC blood counter.

### Histopathology

Organs for histopathological analysis were fixed in 4% formaldehyde (Genta Medical, UK) followed by paraffin embedding and sectioning. Sections were stained with Hematoxylin-Eosin (Merck) for microscopic examination. FACS-purified erythroid precursors were transferred onto slides using a cytospin centrifuge and stained with May-Grünwald and Giemsa solutions (Merck). Morphological examination was performed using AxioImager Z2 Upright Wide-field Microscope (Zeiss).

### Flow cytometry

We isolated bone marrow cells by crushing hips, femurs and tibias in PBS (Thermo Fisher Scientific) supplemented with fetal calf serum (FCS; 2%; Thermo Fisher Scientific) and EDTA (2 mM; Thermo Fisher Scientific). Isolated cells were filtered through a 70 μm cell strainer (Thermo Fisher Scientific). Antibody labelling was performed in PBS (+2% FCS) for 30 min on ice. Thiazole orange (5 μM; Biotium) was included during antibody labelling where specified. Antibodies are listed in the Supplementary Table 1. Erythrocytes were removed from peripheral blood by Dextran sedimentation (2% in PBS; Merck) and ACK lysis buffer (Thermo Fisher Scientific) before antibody labelling. Experiments were performed using FACSARIA III cell sorter (BD Biosciences) and LSRFortessa flow cytometer (BD Biosciences), and analyzed using FlowJo software (Tree Star, v10.1r7).

### Imaging flow cytometry

Sample preparation was performed as previously described^42^. Briefly, 10 × 10^6^ unfractionated bone marrow cells were fixed using formaldehyde (4%; Alfa Aesar) for 15 min at room temperature. Following two washes with PBS, the cell pellet was cooled on ice for 15 min and permeabilized using ice-cold acetone (a cycle of 50%–100%–50%). Following a wash with PBS (+2% FCS), cells were stained for surface markers. Finally, 10 × 10^6^ cells were resuspended in 100 μL PBS supplemented with DRAQ5 (2.5 μM; BioLegend), with acquisition performed on an ImageStream®X Mark II Imaging Flow Cytometer (Merck) using a ×40 objective lens. Approximately 50,000 events per sample were collected, and data analysis was performed using the associated Image Data Exploration and Analysis software (IDEAS; v.6.2; Merck).

### Cell isolation and culture

c-Kit+ bone marrow cells were enriched using CD117 MicroBeads and MACS separation columns (Miltenyi Biotec), and cultured in OptiMEM I reduced Serum Media (Thermo Fisher Scientific), supplemented with FCS (10%), penicillin/streptomycin (P/S, Life Technologies), β-mercaptoethanol (50 μM; Thermo Fisher Scientific), murine stem cell factor (mSCF; 100 ng/mL, PeproTech), murine interleukin 3 (mIL-3; 10 ng/mL, PeproTech) and murine granulocyte-colony stimulating factor (mG-CSF; 10 ng/mL, PeproTech) ± Dox (1 μg/mL; Merck). Biotinylated antibodies and Anti-Biotin MicroBeads (Miltenyi) were used for lineage depletion. In vitro erythroid culture was performed as previously described^47^. Briefly, 1–2.5 × 10^5^ CFU-E/proerythroblasts isolated from Dox-treated mice were seeded on fibronectin-coated (2 μg/mL; Merck) 48-well plates in Iscove’s modified Dulbecco’s medium (IMDM; Thermo Fisher Scientific) containing FCS (15%), bovine serum albumin (BSA; 1%; Stem Cell Technologies), mSCF (10 ng/mL), recombinant human erythropoietin (10 U/mL; Cell Signaling Technology), human recombinant insulin (100 μg/mL; Merck), recombinant human insulin-like growth factor 1 (hIGF1; 100 ng/mL; Thermo Fisher Scientific), holo-transferrin (200 μg/mL; Merck), L-glutamine (2 mM; Merck), β-mercaptoethanol (50 μM) and P/S. The following day, culture media was replaced with differentiation media consisting of IMDM, FCS (20%), β-mercaptoethanol (50 μM), P/S and L-glutamine (2 mM). Homoharringtonine-supplemented differentiation media was used to assess the enucleation of prospectively purified orthochromatic erythroblasts.

### Transplantation assays

Non-competitive transplantations were performed by injecting 5 × 10^6^ freshly isolated unfractionated bone marrow cells in 250 μL PBS (+2% FCS) into the tail vein of lethally irradiated (2 × 500 cGY) congenic (CD45.1) wild-type recipients. Reconstituted mice were allowed to recover for 2 weeks before Dox administration.

### Protein synthesis rate measurement

O-propargyl-puromycin (OP-puro) labelling experiments were performed as previously described^48^. Briefly, OP-puro (50 mg/kg in 200 μL PBS; Jena Bioscience) was injected intraperitoneally and bone marrow cells were isolated after 1 h. 3 × 10^6^ cells were fixed with formaldehyde (4%) for 15 min at room temperature. Following two washes with PBS (+2% FCS), cells were stained with antibodies against cell surface markers. Stained cells were permeabilized using PBS supplemented with saponin (0.1%; Merck) and FCS (2%). The Click reaction was performed using the Click-iT™ Plus OPP Alexa Fluor™ 488 Protein Synthesis Assay Kit according to the manufacturer’s instructions (Thermo Fisher Scientific).

### Polysome profiling experiments

Equal numbers of c-Kit+ bone marrow cells were expanded in the presence of doxycycline for 24 h and treated with cycloheximide (CHX; 100 μg/mL; Merck) for 8 min at 37 °C before harvesting by centrifugation. Cells were washed twice with ice-cold PBS supplemented with CHX (100 μg/mL) and lysed for 30 min on ice in ‘standard’ lysis buffer (20 mM Hepes pH 7.5, 50 mM KCl, 10 mM Mg(CH_3_COO)_2_, 100 μg/mL CHX, cOmplete™ EDTA-free Protease Inhibitor Cocktail (Merck), RNaseOUT recombinant ribonuclease inhibitor (200 U/mL; Thermo Fisher Scientific) and IGEPAL® CA-630 (0.5%; Merck), Dithiothreitol (DTT; 2 mM; Merck). The lysate was cleared by centrifugation (18,000 × *g* for 8 min at 4 °C) and loaded onto a 5–45% (w/v) sucrose gradient (prepared in 20 mM Hepes pH 7.5, 50 mM KCl, 10 mM Mg(CH_3_COO)_2_, 100 μg/mL CHX and cOmplete™ EDTA-free Protease Inhibitor Cocktail) prepared in a polypropylene centrifuge tube (14 × 95 mm; Beckman Coulter). A Gradient Master (Biocomp) was used to prepare the sucrose gradients. After centrifugation (285,000 × g for 2 h at 4 °C using a Beckman SW40Ti rotor), polysome profiles were recorded using an Äktaprime plus chromatography system (GE Healthcare). Proteins were precipitated with trichloroacetic acid (25% (vol/vol); 15 min on ice). Following centrifugation at 18,000 × *g* for 5 min at 4 °C, protein precipitates were washed with ice-cold acetone. After drying at room temperature, protein pellets were resuspended in 1× NuPAGE LDS Sample Buffer (Thermo Fisher Scientific). Vacant 80S monosomes were dissociated in a high salt lysis buffer (20 mM Hepes pH 7.5, 200 mM KCl, 10 mM Mg(CH_3_COO)_2_, 100 μg/mL CHX, cOmplete™ EDTA-free Protease Inhibitor Cocktail (Merck), 200 U/mL RNaseOUT, 0.5% IGEPAL® CA-630 and 2 mM DTT), and the lysate was loaded onto a 5–45% (w/v) sucrose gradient prepared in 20 mM Hepes pH 7.5, 200 mM KCl, 10 mM Mg(CH_3_COO)_2_, 100 μg/mL CHX and cOmplete™ EDTA-free Protease Inhibitor Cocktail. Ribosomal subunit quantification was performed using a magnesium-free lysis buffer (20 mM Hepes pH 7.5, 50 mM KCl, 100 μg/mL CHX, cOmplete™ EDTA-free Protease Inhibitor Cocktail, RNaseOUT recombinant ribonuclease inhibitor (200 U/mL), IGEPAL® CA-630 (0.5%) and DTT (2 mM). The lysate was cleared by centrifugation (18,000 × *g* for 8 min at 4 °C) and loaded onto a 5–45% (w/v) sucrose gradient (prepared in 20 mM Hepes pH 7.5, 50 mM KCl, 100 μg/mL CHX and cOmplete™ EDTA-free Protease Inhibitor Cocktail).

### Purification of recombinant SBDS and EFL1 proteins

SBDS and EFL1 proteins were purified as previously described^13^.

### eIF6 release assay

Preparation of mature 80S ribosomes—Expanded mouse c-Kit+ bone marrow cells were lysed in ‘standard’ lysis buffer (20 mM Hepes pH 7.5, 50 mM KCl, 10 mM Mg(CH_3_COO)_2_, supplemented with cOmplete™ EDTA-free Protease Inhibitor Cocktail, 200 U/mL RNaseOUT inhibitor, 0.5% IGEPAL® CA-630 and 2 mM DTT). A total of 150 A_260_ units of lysate was loaded on six 5–45% (w/v) sucrose gradients, and fractions corresponding to 80 S monosomes were collected and further concentrated by centrifuging 30 min at 80000 g in a Beckman MLA-80 rotor fitted in an Optima MAX-XP ultracentrifuge. The sedimented 80 S particles were resuspended in ‘standard’ buffer and aliquots stored at −80 °C.

Preparation of exogenous eIF6—c-Kit+ bone marrow cells isolated from transgenic eIF6 mice (genotype [*M2-rtTA/M2-rtTA*][*EIF6*/+]) were expanded and treated with doxycycline for 24 h to induce eIF6 overexpression. Cells were then harvested and lysed in ‘dissociation’ lysis buffer (20 mM Hepes pH 7.5, 500 mM KCl, 2 mM Mg(CH_3_COO)_2_ supplemented with 100 μg/mL CHX, cOmplete™ EDTA-free Protease Inhibitor Cocktail, 200 U/mL RNaseOUT inhibitor, 0.5% IGEPAL® CA-630 and 2 mM DTT). A total of 29 A_260_ units of lysate was run on a single 5–45% (w/v) sucrose gradient and the free fraction, which contains the vast majority of cellular eIF6 but is devoid of ribosomes, was collected and aliquots stored at −80 °C.

In vitro eIF6 release assay—In the first part of the assay, 10 μL (1.25 A_260_ units) of mature 80S particles were mixed with 100 μL of exogenous eIF6 in ‘dissociation’ buffer. The reaction mix was then incubated at 37 °C for 15 min both to promote the dissociation of the mature 80S particles into 40S and 60S subunits, and to allow the binding of the exogenous eIF6 to 60S subunits. The amount of eIF6 supplied was optimized empirically to be in slight excess over 60S subunits, thus saturating the available 60S subunits without a significant accumulation in the free fraction. In the second part of the protocol, the reaction mix was diluted with 500 μL of prewarmed KCl-free buffer (20 mM Hepes pH 7.5, 10 mM Mg(CH_3_COO)_2_), and incubated at 37 °C for 5 min to allow reassembly of 80S particles. Since the joining of 40S and 60S subunits into 80S particles is proportional to the release of eIF6 from 60S subunits, this experimental strategy allows the assessment of eIF6 release based on quantification of 80S to 60S ratio. The reaction mix was split equally into two tubes that were supplied either with 1 mM GTP or 1 mM GTP + 1250 nM SBDS + 600 nM EFL1. Following 1 h incubation at 25 °C, the reaction mixes were cooled down on ice, and loaded on 5–45% (w/v) sucrose gradients prepared in ‘standard’ buffer conditions.

### Electron cryo-microscopy sample preparation and data collection

c-Kit+ bone marrow cells were isolated from control mice that do not harbor *EIF6* transgene and expanded keeping cell concentration below one million cells per mL. CHX-treated cells were lysed in ‘standard’ lysis buffer as described in ‘Polysome profiling experiments’. Following sucrose gradient sedimentation (5–45% (w/v) gradient prepared in 20 mM Hepes pH 7.5, 50 mM KCl, 10 mM Mg(CH_3_COO)_2_, 100 μg/mL CHX and cOmplete™ EDTA-free Protease Inhibitor Cocktail), 60S subunits from multiple gradients were pooled, sedimented by centrifugation (45 min at 80,000 × *g* in an MLA-80 rotor), resuspended in 20 mM HEPES pH 7.5, 50 mM KCl, 5 mM Mg(CH_3_COO)_2_ at a concentration of 100 mM, and stored at −80 °C. 60S ribosomal subunits were thawed on ice and centrifuged in a benchtop centrifuge for 10 min at 20,000 × *g* and the supernatant was carefully recovered. EM grids were prepared by depositing 3 μl of 60S subunits at 100 nM to freshly glow-discharged (PELCO easyGlow) Quantifoil R 1.2/1.3 holey carbon grids (Quantifoil Micro Tools GmbH). Grids were then blotted with a Vitrobot Mark IV (FEI company) using the following parameters: blot time 1 s, blot force −7, wait time 10 s, no drain time. Blotted grids were finally vitrified in liquid ethane and stored in liquid nitrogen. Grids were screened on a Tecnai T12 microscope (FEI Company) and data acquisition performed under low-dose conditions on a Titan Krios microscope (FEI Company) operated at 300 kV over 24 h. The dataset was recorded on a Falcon III detector (FEI Company) at a nominal magnification of ×75,000 (effective pixel size of 1.10 Å on the object scale) with a defocus range of −0.8 to −3.2 μm and a total dose of ~77 e−/Å^2^ accumulated over 2 s exposures in 38 fractions. The acquisition of 3024 movies was performed semi-automatically using EPU software (FEI Company).

### Electron cryo-microscopy data processing

Data processing was handled within the RELION software package^49–51^. Movies were first corrected for motion using Motioncor2^52^ and CTF was estimated by CTFFIND4^53^. Particles were then picked using the Laplacian-of-Gaussian (LoG) filter, extracted and 2D classified in RELION. 3D classification was then performed on selected 2D classes to further discard non-ribosomal and contaminating 80S particles. The 60S ribosomal subunit-containing class was selected for 3D auto-refinement to generate a consensus map. Masking and auto-sharpening was done through post-processing in RELION to obtain the final high-resolution map.

To quantify the proportion of eIF6-bound ribosomal particles, we made use of a combination of particle subtraction and 3D masked classifications in RELION (Fig. 1b). We first focused on the L1-stalk to sort particles relative to their maturation state. We generated a mask around the L1-stalk and the tRNA E-site from the consensus map and used it in 3D masked classification, leading to the isolation of mature ribosomal particles (88% of consensus-refined particles). We then generated a soft-edged mask around the area of the eIF6 binding site from the consensus map as an input. Signal outside this mask was subtracted in the newly obtained mature ribosomal particles subset. We finally generated 3D classes focusing on the area inside the mask. 4 classes were obtained, of which 3 showed clear density inside the masked area indicating the unequivocal presence of eIF6 and were then pooled for quantification (83% of mature ribosomal particles).

### Immunoblotting

Proteins in 1× NuPAGE LDS sample buffer (with 50 mM DTT) were incubated at 80 °C for 10 min and run on NuPAGE Bis-Tris polyacrylamide gels in NuPAGE MOPS SDS running buffer (Thermo Fisher Scientific). The iBlot 2 gel transfer device (Thermo Fisher Scientific) was used to transfer proteins to nitrocellulose membranes. Membranes were blocked in 5% milk in PBS supplemented with 0.1% tween (NBS Biologicals) for 1 h, and subsequently incubated with appropriate primary antibodies overnight at 4 °C on a shaker. After 3 × 10 min washes with PBS-tween, the blots were incubated with the appropriate secondary horseradish peroxidase-conjugated antibody at room temperature for 1 h followed by detection using the SuperSignal West Pico PLUS reagents (Thermo Fisher Scientific). For a full list of antibodies, see Supplementary Table 2. ImageJ 1.49 v was used to quantify eIF6 protein abundance.

### Subcellular fractionation

1 × 10^6^ freshly isolated bone marrow cells from Dox-treated mice were washed twice with ice-cold PBS and resuspended in 0.5 mL of ‘standard’ lysis buffer (20 mM Hepes pH 7.5, 50 mM KCl, 10 mM Mg(CH_3_COO)_2_, cOmplete™ EDTA-free Protease Inhibitor Cocktail, 0.5% IGEPAL® CA-630, and 2 mM DTT. Following 30 min incubation on ice, lysates were centrifuged for 5 min at 7000 × *g* to pellet the nuclei, while supernatants representing the cytosolic fraction were collected. Following a wash with ice-cold PBS and centrifugation as above, the nuclear pellet was resuspended in 0.5 mL of ice-cold RIPA buffer supplied with 1 U/mL Benzonase (Merck), and incubated on a rotator for 1 h at 4 °C. Following centrifugation for 5 min at 7000 × *g* to pellet insoluble material, supernatant representing the nuclear fraction was collected. Finally, both fractions were resuspended in 1x NuPAGE LDS sample buffer with 50 mM DTT.

### Quantitative real-time PCR

Total RNA was isolated from FACS-purified cells using the RNeasy mini kit (Qiagen). cDNA was transcribed with SuperScript III reverse trancriptase (Thermo Fisher Scientific). Real-time PCR reactions were performed using the SsoFast™ EvaGreen^®^ Supermix (Bio-Rad) and ABI 7900HT fast Real-time PCR system (Thermo Fisher Scientific). Primers are listed in Supplementary Table 3.

### Light microscopy

*Drosophila* were maintained using standard culture techniques. All crosses were performed at 25 °C. Fly strains and genotypes are described in Supplementary Tables 4 and 5. Whole *Drosophila* samples were collected at 1, 3, 5, and 11 days after egg laying (AEL). Larvae were fixed with 4% paraformaldehyde and adult flies were frozen before photography. For the imaging of fly eyes, two to four day old *Drosophila* adults were frozen at −20 °C for 1 h. Both whole fly and adult eye photographs were collected using a Nikon SMZ18 microscope with NIS-Elements D (version 4.40).

### Scanning electron microscopy

*Drosophila* adult eye samples were prepared as described^54^. Samples were viewed on a Philips XL30 scanning electron microscope.

### Immunostaining

*Drosophila* wing discs dissected from third instar larvae in culture medium (*Drosophila* M3 media (Sigma), 10% FCS (Sigma) and P/S (Sigma) were collected within 10 min into culture medium containing 50 μM of OP-Puro (Invitrogen) and kept in a 25 °C incubator for 30 min. Wing discs were then washed twice with ice-cold PBS (Invitrogen) with 1% BSA (Sigma) and 100 μg/ml CHX (Sigma). Wing discs were fixed and permeabilized using the Cytofix/Cytoperm Fixation Permeabilization Kit (BD Biosciences). Azide-alkyne cycloaddition was performed using the Click-iT Cell Reaction Buffer Kit (Invitrogen) with azide conjugated to Alexa Fluor 596 at 5 μM final concentration. Following a 30 min reaction, wing discs were washed three times in PBS and mounted on slides in medium containing DAPI (Vector). Images were collected on a Zeiss LSM710 confocal system and imported to Image J 2.1.0 v and Photoshop (Adobe) and adjusted for brightness and contrast uniformly across entire fields.

### Sucrose gradient sedimentation of *Drosophila* cell extracts

Ribosomal subunits from *Drosophila* second instar larval cells were fractionated by sucrose density gradient sedimentation as previously described^15^.

### Statistics

Student’s *t* test was used to determine statistical significance. Two-tailed *P* values are shown. All graphs are presented as mean ± standard deviation.

### Data analysis

Graphs were prepared using Prism 8 and 9 (Graphpad) and Photoshop CS5 (Adobe). Figures were prepared using Illustrator CS6 (Adobe).

### Reporting summary

Further information on research design is available in the Nature Research Reporting Summary linked to this article.

## Supplementary information

Supplementary Information

Reporting Summary

## Data Availability

The data that support this study are available from the corresponding author upon reasonable request. Source data are provided with this paper.

## Source data

Source data

## References

[1] Ceci M (2003). Release of eIF6 (p27BBP) from the 60S subunit allows 80S ribosome assembly. Nature.

[2] Russell DW, Spremulli LL (1980). Mechanism of action of the wheat germ ribosome dissociation factor: interaction with the 60 S subunit. Arch. Biochem. Biophys..

[3] Gartmann M (2010). Mechanism of eIF6-mediated inhibition of ribosomal subunit joining. J. Biol. Chem..

[4] Basu U, Si K, Warner JR, Maitra U (2001). The Saccharomyces cerevisiae TIF6 gene encoding translation initiation factor 6 is required for 60S ribosomal subunit biogenesis. Mol. Cell Biol..

[5] Senger B (2001). The nucle(ol)ar Tif6p and Efl1p are required for a late cytoplasmic step of ribosome synthesis. Mol. Cell.

[6] Becam AM, Nasr F, Racki WJ, Zagulski M, Herbert CJ (2001). Ria1p (Ynl163c), a protein similar to elongation factors 2, is involved in the biogenesis of the 60S subunit of the ribosome in *Saccharomyces cerevisiae*. Mol. Genet. Genomics.

[7] Menne TF (2007). The Shwachman-Bodian-Diamond syndrome protein mediates translational activation of ribosomes in yeast. Nat. Genet..

[8] Finch AJ (2011). Uncoupling of GTP hydrolysis from eIF6 release on the ribosome causes Shwachman-Diamond syndrome. Genes Dev..

[9] Wong CC, Traynor D, Basse N, Kay RR, Warren AJ (2011). Defective ribosome assembly in Shwachman-Diamond syndrome. Blood.

[10] Weis F (2015). Mechanism of eIF6 release from the nascent 60S ribosomal subunit. Nat. Struct. Mol. Biol..

[11] Kargas, V. et al. Mechanism of completion of peptidyltransferase centre assembly in eukaryotes. *Elife* **8**, e44904 (2019). 10.7554/eLife.44904 PMC6579518 31115337

[12] Boocock GR (2003). Mutations in SBDS are associated with Shwachman-Diamond syndrome. Nat. Genet.

[13] Tan S (2019). EFL1 mutations impair eIF6 release to cause Shwachman-Diamond syndrome. Blood.

[14] Warren AJ (2018). Molecular basis of the human ribosomopathy Shwachman-Diamond syndrome. Adv. Biol. Regul..

[15] Tan S (2021). Somatic genetic rescue of a germline ribosome assembly defect. Nat. Commun..

[16] Kennedy AL (2021). Distinct genetic pathways define pre-malignant versus compensatory clonal hematopoiesis in Shwachman-Diamond syndrome. Nat. Commun..

[17] Shen PS (2015). Protein synthesis. Rqc2p and 60S ribosomal subunits mediate mRNA-independent elongation of nascent chains. Science.

[18] Su T (2019). Structure and function of Vms1 and Arb1 in RQC and mitochondrial proteome homeostasis. Nature.

[19] Alkalaeva EZ, Pisarev AV, Frolova LY, Kisselev LL, Pestova TV (2006). In vitro reconstitution of eukaryotic translation reveals cooperativity between release factors eRF1 and eRF3. Cell.

[20] Pisarev AV (2010). The role of ABCE1 in eukaryotic posttermination ribosomal recycling. Mol. Cell.

[21] Shoemaker CJ, Green R (2011). Kinetic analysis reveals the ordered coupling of translation termination and ribosome recycling in yeast. Proc. Natl Acad. Sci. USA.

[22] Skabkin MA (2010). Activities of Ligatin and MCT-1/DENR in eukaryotic translation initiation and ribosomal recycling. Genes Dev..

[23] Ban N (2014). A new system for naming ribosomal proteins. Curr. Opin. Struct. Biol..

[24] Heuer A (2017). Structure of the 40S-ABCE1 post-splitting complex in ribosome recycling and translation initiation. Nat. Struct. Mol. Biol..

[25] Tzamarias D, Roussou I, Thireos G (1989). Coupling of GCN4 mRNA translational activation with decreased rates of polypeptide chain initiation. Cell.

[26] Ashe MP, De Long SK, Sachs AB (2000). Glucose depletion rapidly inhibits translation initiation in yeast. Mol. Biol. Cell.

[27] Surks MI, Berkowitz M (1971). Rat hepatic polysome profiles and in vitro protein synthesis during hypoxia. Am. J. Physiol..

[28] Uesono Y, Toh EA (2002). Transient inhibition of translation initiation by osmotic stress. J. Biol. Chem..

[29] Pisareva VP, Skabkin MA, Hellen CU, Pestova TV, Pisarev AV (2011). Dissociation by Pelota, Hbs1 and ABCE1 of mammalian vacant 80S ribosomes and stalled elongation complexes. EMBO J..

[30] Mills EW, Wangen J, Green R, Ingolia NT (2016). Dynamic regulation of a ribosome rescue pathway in erythroid cells and platelets. Cell Rep..

[31] Groft CM, Beckmann R, Sali A, Burley SK (2000). Crystal structures of ribosome anti-association factor IF6. Nat. Struct. Biol..

[32] Klinge S, Voigts-Hoffmann F, Leibundgut M, Arpagaus S, Ban N (2012). Crystal structure of the eukaryotic 60S ribosomal subunit in complex with initiation factor 6. Science.

[33] Yip MCJ (2019). Mechanism for recycling tRNAs on stalled ribosomes. Nat. Struct. Mol. Biol..

[34] Beard C, Hochedlinger K, Plath K, Wutz A, Jaenisch R (2006). Efficient method to generate single-copy transgenic mice by site-specific integration in embryonic stem cells. Genesis.

[35] Urlinger S (2000). Exploring the sequence space for tetracycline-dependent transcriptional activators: novel mutations yield expanded range and sensitivity. Proc. Natl Acad. Sci. USA.

[36] Martin TE, Hartwell LH (1970). Resistance of active yeast ribosomes to dissociation by KCl. J. Biol. Chem..

[37] Lee, S. et al. Somatic uniparental disomy mitigates the most damaging EFL1 allele combination in Shwachman-Diamond syndrome. *Blood* **138**, 2117–2128 (2021). 10.1182/blood.2021010913 34115847

[38] Pronk CJ (2007). Elucidation of the phenotypic, functional, and molecular topography of a myeloerythroid progenitor cell hierarchy. Cell Stem Cell.

[39] Chen K (2009). Resolving the distinct stages in erythroid differentiation based on dynamic changes in membrane protein expression during erythropoiesis. Proc. Natl Acad. Sci. USA.

[40] Satake S (2012). C/EBPβ is involved in the amplification of early granulocyte precursors during candidemia-induced “emergency” granulopoiesis. J. Immunol..

[41] McGrath KE, Catherman SC, Palis J (2017). Delineating stages of erythropoiesis using imaging flow cytometry. Methods.

[42] Konstantinidis DG (2012). Signaling and cytoskeletal requirements in erythroblast enucleation. Blood.

[43] Jarzebowski L (2018). Mouse adult hematopoietic stem cells actively synthesize ribosomal RNA. RNA.

[44] Gandin V (2008). Eukaryotic initiation factor 6 is rate-limiting in translation, growth and transformation. Nature.

[45] Zhang S, Shi M, Hui CC, Rommens JM (2006). Loss of the mouse ortholog of the shwachman-diamond syndrome gene (Sbds) results in early embryonic lethality. Mol. Cell Biol..

[46] Tourlakis ME (2012). Deficiency of Sbds in the mouse pancreas leads to features of Shwachman-Diamond syndrome, with loss of zymogen granules. Gastroenterology.

[47] Shuga J, Zhang J, Samson LD, Lodish HF, Griffith LG (2007). In vitro erythropoiesis from bone marrow-derived progenitors provides a physiological assay for toxic and mutagenic compounds. Proc. Natl Acad. Sci. USA.

[48] Signer RA, Magee JA, Salic A, Morrison SJ (2014). Haematopoietic stem cells require a highly regulated protein synthesis rate. Nature.

[49] Scheres SH, Chen S (2012). Prevention of overfitting in cryo-EM structure determination. Nat. Methods.

[50] Scheres SH (2012). RELION: implementation of a Bayesian approach to cryo-EM structure determination. J. Struct. Biol..

[51] Kimanius, D., Forsberg, B. O., Scheres, S. H. & Lindahl, E. Accelerated cryo-EM structure determination with parallelisation using GPUs in RELION-2. *Elife* **5**, e18722 (2016). 10.7554/eLife.18722 PMC5310839 27845625

[52] Zheng SQ (2017). MotionCor2: anisotropic correction of beam-induced motion for improved cryo-electron microscopy. Nat. Methods.

[53] Rohou A, Grigorieff N (2015). CTFFIND4: Fast and accurate defocus estimation from electron micrographs. J. Struct. Biol..

[54] Tan SJ, Amos W, Laughlin SB (2005). Captivity selects for smaller eyes. Curr. Biol..

