# Supplementary material for: eIF6 rebinding dynamically couples ribosome maturation and translation
Source: Nat Commun. 2022 Mar 23;13:1562. doi: 10.1038/s41467-022-29214-7 (PMC8943182; doi:10.1038/s41467-022-29214-7)

Supplementary Information

eIF6 rebinding dynamically couples ribosome maturation and translation

Jaako *et al*.

SUPPLEMENTARY FIGURE LEGENDS

Supplementary Figure 1. Fourier shell correlation curves of the cryo-EM data set. Fourier shell correlation curves of both solvent masked and unmasked final maps indicating maximum resolution at 0.143 'gold standard' threshold.

Supplementary Figure 2. Genetic interaction between eIF6 and Sbds in *Drosophila* larval development

(a) Second instar larval extracts from indicated genotypes were immunoblotted to visualise the indicated proteins. The additional 30 kDa band detected by the eIF6 antibody represents the overexpressed FLAG-tagged eIF6. One representative of two independent experiments is shown.

(b) Extracts from *Drosophila* second instar larvae with the indicated genotypes were fractionated by sucrose gradient sedimentation. One representative out of three independent experiments is shown.

(c) Immunoblots of the fractionated sucrose gradients shown in (b) to visualise the indicated proteins. One representative out of two independent experiments is shown.

Supplementary Figure 3. Genetic interaction between eIF6 and Sbds in *Drosophila* eye development.

Representative scanning electron microscopy images showing the *Drosophila* eye phenotypes in the indicated genotypes (n=3 biologically independent samples).

Supplementary Figure 4. Dox administration has no acute impact on weight gain in eIF6^{hi} mice. Time represents weeks fed on doxycycline diet. Uninduced and 1 week, n=12 and 11 animals per genotype; 16 weeks, n=12 and 10 animals per genotype; 54

weeks, n=11 and 12 animals per genotype. Data are presented as mean +/- standard deviation. Student's *t* test was used to determine statistical significance. Two-tailed *P* values are shown.

Supplementary Figure 5. Validation of *EIF6* transgene expression in eIF6^{hi} mice.

(a) Quantitative real-time PCR analysis of *EIF6* mRNA expression in LSK, preGM/GMP and preCFU-E/CFU-E progenitor cells isolated from adult mice after two weeks of doxycycline administration. (n=4 independent biological samples per genotype).

(b) Sucrose gradient sedimentation of extracts from cultured c-Kit+ bone marrow cells derived from the indicated mouse strains. Dox induction, 24 h.

(c) Immunoblotting analysis to visualise the distribution of eIF6, Sbds, uL5 and eS19 across the sucrose density gradients shown in (b).

(d) Subcellular fractionation of freshly isolated unfractionated bone marrow cells from adult mice after two weeks of doxycycline administration. C= cytoplasmic fraction, N= nuclear fraction. LSK=Lineage- Sca-1+ c-Kit+, preGM= pre-granulocyte-macrophage progenitor, GMP= granulocyte-macrophage progenitor, pre-CFU-E= pre-colony-forming unit-erythroid progenitor. All graphs show mean +/- standard deviation. Student's *t* test was used to determine statistical significance. Two-tailed *P* values are shown.

Supplementary Figure 6. Peripheral blood analysis of eIF6^{hi} mice after two weeks of doxycycline induction. 2 and 20 weeks, n=13 and 15 animals per genotype; 1 year, n=12 and 13 animals per genotype. All graphs show mean +/- standard deviation.

Student’s *t* test was used to determine statistical significance. Two-tailed *P* values are shown.

Supplementary Figure 7. Histological examination of the eIF6^{hi} mice.

(a) Representative bone marrow sections (40X).

(b) Spleen weight (n=7 animals per genotype).

(c) Representative spleen sections (40X).

Data are presented as mean values +/- SD. Student’s *t* test was used to determine statistical significance. Two-tailed *P* values are shown.

Supplementary Figure 8. Flow cytometry strategy to quantify bone marrow subpopulations.

Supplementary Figure 9. Flow cytometric analysis of the haematopoietic phenotype of the eIF6^{hi} mice.

(a) Bone marrow cellularity and

(b) frequency of haematopoietic stem and progenitor cells in the bone marrow after two weeks of Dox administration (n=7 independent biological samples per genotype).

(c) Frequency of erythroid precursor cells in the spleen after two weeks of Dox administration (n=7 independent biological samples per genotype).

(d) I*n vitro* differentiation culture of prospectively purified CFU-E/proerythroblast cells. One representative out of two independent experiments is shown. Fresh cells were isolated from the bone marrow of Dox-treated eIF6^{hi} mice, and depleted for TER-119, Gr-1, CD11b, CD4, CD8, B220, CD41, CD16/32, CD150 and Sca-1. Enriched cells were cultured with or without Dox.

HSC = haematopoietic stem cell; MPP = multipotent progenitor; pGM= pre-granulocyte-macrophage progenitor; GMP = granulocyte-macrophage progenitor, preMegE = pre-megakaryocyte-erythroid progenitor, MkP = megakaryocyte progenitor, preCFU-E = pre-colony-forming unit-erythroid progenitor. All graphs show mean +/- standard deviation. Student's *t* test was used to determine statistical significance. Two-tailed *P* values are shown.

Supplementary Figure 10. Flow cytometry quantification of granulocyte precursors in the bone marrow.

(a) Flow cytometry strategy.

(b) Frequency of myeloid progenitors and granulocyte precursors in the bone marrow after two weeks of Dox administration (n=7 independent biological samples per genotype). Student's *t* test was used to determine statistical significance. Two-tailed *P* values are shown. Data are presented as mean values +/- SD. pGM= pre-granulocyte-macrophage progenitor; GMP = granulocyte-macrophage progenitor.

Supplementary Figure 11. IDEAS strategy to visualise enucleating erythroblasts in the bone marrow.

Supplementary Figure 12. The haematopoietic phenotype in eIF6^{hi} mice is autonomous to the blood system.

(a) Overview of the transplantation strategy. Five million freshly isolated unfractionated bone marrow cells from uninduced control or eIF6^{hi} mice were transplanted into the tail vein of lethally irradiated (2x 500 cGy) wild-type recipients (CD45.1). Two weeks after transplantation, Dox was administered to the recipient mice

to induce expression of the *EIF6* transgene, and peripheral blood cellularity was analysed at indicated time-points.

(b) The number of erythrocytes and platelets, hemoglobin concentration and mean corpuscular volume (MCV) in the peripheral blood over time (n=9 and 10 animals per genotype).

(c) Donor cell reconstitution within the different white blood cell lineages after 16 weeks of Dox administration (n=9 and 10 animals per genotype). All graphs show mean +/- standard deviation. Student's *t* test was used to determine statistical significance. Two-tailed *P* values are shown.

Supplementary Table 1. Antibodies and reagents used in flow cytometry.

	Fluorochrome	Cat#	Manufacturer	Dilution
<u>Bone marrow analysis</u>				
CD71	FITC	113806	Biolegend	1:200
CD44	FITC	553133	BD Biosciences	1:200
CD48	FITC	103404	Biolegend	1:200
CD41	PE	12-0411-83	eBioscience	1:200
CD45.1	PE	110708	Biolegend	1:200
GR1	PE-Cy5 (Lineage)	108410	Biolegend	1:400
CD11b	PE-Cy5 (Lineage)	101210	Biolegend	1:400
B220	PE-Cy5 (Lineage)	103210	Biolegend	1:400
CD3	PE-Cy5 (Lineage)	100310	Biolegend	1:400
Ter119	PE-Cy5 (Lineage)	116210	Biolegend	1:400
Ter119	PE-Cy7	25-5921-82	eBioscience	1:400
CD16/32	PE-Cy7	101317	Biolegend	1:200
CD150	APC	115910	Biolegend	1:200
c-Kit	APC-eFluor780	47-1171-82	eBioscience	1:100
Endoglin	Biotin	120404	Biolegend	1:200
Sca-1	Pacific blue	122520	Biolegend	1:200
CD44	Pacific blue	103019	Biolegend	1:200
CD71	BV421	113813	Biolegend	1:200
Ly-6G	FITC	127605	Biolegend	1:200
Streptavidin	QD605	Q10101MP	Life Technologies	1:200

Peripheral blood analysis

CD45.2	FITC	109806	Biolegend	1:200
CD45.1	PE	110708	Biolegend	1:200
CD19	PE-Cy7	25-0193-82	eBioscience	1:200
CD11b	APC	101212	Biolegend	1:800
CD3	Alexa Fluor® 700	100216	Biolegend	1:400

Lineage depletion/enrichment

CD71	Biotin	113803	Biolegend	1:200
Ter119	Biotin	116204	Biolegend	1:200
CD4	Biotin	100404	Biolegend	1:200
CD8a	Biotin	100704	Biolegend	1:200
B220	Biotin	103204	Biolegend	1:200
CD16/32	Biotin	101303	Biolegend	1:200
CD41	Biotin	133930	Biolegend	1:200
Sca-1	Biotin	108103	Biolegend	1:200
Gr-1	Biotin	108404	Biolegend	1:200
CD11b	Biotin	101204	Biolegend	1:200

Supplementary Table 2. Antibodies used in immunoblotting.

	**Cat#**	**Manufacturer**	**Dilution**
Primary antibodies			
EIF6	GTX117971	GeneTex	1:1000
eS6 (Rps6)	2317S	CST	1:800
FLAG	ab1257	Abcam	1:10000
GAPDH	G9545	Merck	1:10000
NMD3	16060-1-AP	Proteintech	1:1000
eS19 (Rps19)	15085-1-AP	Proteintech	1:1000
uL5 (Rpl11)	16277-1-AP	Proteintech	1:1000
eL8 (Rpl7a)	2415	CST	1:1000
SBDS	GTX109168	GeneTex	1:1000
Sbds (*Drosophila*)	Tan, S[15]	Warren Lab[15]	1:1000
uL14 (Rpl23)	ab112587	Abcam	1:1000
Lamin B1	12987-1-AP	Proteintech	1:2000
Secondary antibodies			
Anti-rabbit IgG, HRP	7074	CST	1:5000
Anti-mouse IgG, HRP	A5287	Sigma	1:10000
Anti-goat IgG, HRP	sc-2020	Santa Cruz	1:10000

Supplementary Table 3. Custom primers used for quantitative real-time PCR

**Gene**	**Primer**
Actb	5’-CCACAGCTGAGAGGCAAATC-3’
	5’-CTTCTCCAGGGAGGAAGAGG-3’
Eif6	5’-CCAAGTACCATTGCCACCAG-3’
(mouse)	5’-GGAAAATGAGCCAAAGTCCAGAG-3’
EIF6	5’-AATGTCACCACCTGCAATGAC-3’
(Total: mouse + transgene)	5’-TGTCTGAAGACTTCCACCTTGAG-3’

Supplementary Table 4. *Drosophila melanogaster* strains.

Name	Genotype	Source
da-GAL4	w^{*}*; P{GAL4-da.G32}UH1*	Bloomington *Drosophila* Stock Center
$Efl1^{RNAi}$	w^{1118}*; P{GD11381}v34884/CyO*	Vienna *Drosophila* RNAi Center
En>GAL4	w^{1118}*; P{en2.4-GAL4}e16E, P{UAS-2xEGFP}AH2*	Bloomington *Drosophila* Stock Center
GMR-GAL4	w^{*}*; P{GAL4-ninaE.GMR}12*	M. Freeman
$Sbds^{RNAi}$	w^{1118}*;; pUAS-8549R-4*	NIG-Fly
UAS-eIF6	w^{1118}*;; pUAS-eIF6-FLAG*	A.J. Warren
UAS-Sbds	w^{1118}*;; pUAS-Sbds-FLAG*	A.J. Warren

Supplementary Table 5. ***Drosophila melanogaster*** **genotypes.**

**Figure**	**Name**	**Genotype**
Fig 3a	Control	w^{1118};; *da-GAL4*
Fig 3a	$Sbds^{RNAi}$/+	w^{1118};; *da-GAL4/*$Sbds^{RNAi}$
Fig 3a	*eIF6/+*	w^{1118};; *da-GAL4/UAS-eIF6*
Fig 3a	*eIF6/+*, $Sbds^{RNAi}$/+	w^{1118};;*da-GAL4/UAS-eIF6,UAS-*$Sbds^{RNAi}$
Fig 3b, Suppl Fig 3	Control	w^{1118}; *GMR-GAL4/+*
Fig 3b, Suppl Fig 3	*eIF6/+*, $Sbds^{RNAi}$/+	w^{1118};*GMR-GAL4/+; UAS-eIF6,UAS-*$Sbds^{RNAi}$/+
Fig 3b, Suppl Fig 3	$Sbds^{RNAi}$/+	w^{1118}; *GMR-GAL4/+; UAS-*$Sbds^{RNAi}$/+
Fig 3b, Suppl Fig 3	*eIF6/+*	w^{1118}; *GMR-GAL4/+; UAS-eIF6/+*
Fig 3b	*eIF6/+*, $Efl1^{RNAi}$/+	w^{1118}; *GMR-GAL4/UAS-*$Efl1^{RNAi}$; *UAS-eIF6/+*
Fig 3b	*eIF6/eIF6*	w^{1118}; *GMR-GAL4/+; UAS-eIF6/UAS-eIF6*
Fig 3b	$Efl1^{RNAi}$/+	w^{1118}; *GMR-GAL4/UAS-*$Efl1^{RNAi}$
Fig 3b	*EES*	w^{1118}; *GMR-GAL4/UAS-*$Efl1^{RNAi}$; *UAS-eIF6,UAS-*$Sbds^{RNAi}$/+[i]
Fig 3c	*En>GFP*	w^{1118}; *en2.4-GAL4, UAS-2xeGFP/+*
Fig 3c	*En>GFP, eIF6/+*	w^{1118}; *en2.4-GAL4, UAS-*

		2xeGFP/+; UAS-eIF6/+
Suppl Fig 3	$Sbds^{RNAi}/Sbds^{RNAi}$	w^{1118}*; GMR-GAL4/+; UAS-* $Sbds^{RNAi}/Sbds^{RNAi}$
Suppl Fig 3	*eIF6/+,* $Sbds^{RNAi}$*/Sbds*	w^{1118}*; GMR-GAL4/+; UAS-eIF6,UAS-*$Sbds^{RNAi}$*/UAS-Sbds*

Supplementary Figure 1

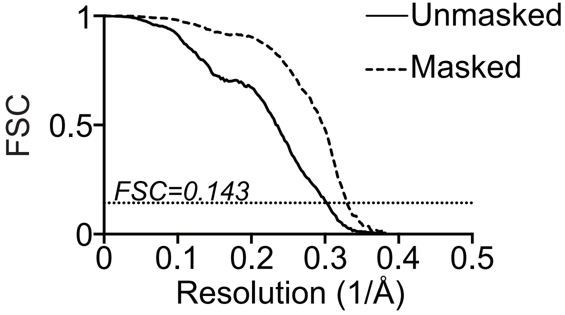

Supplementary Figure 2

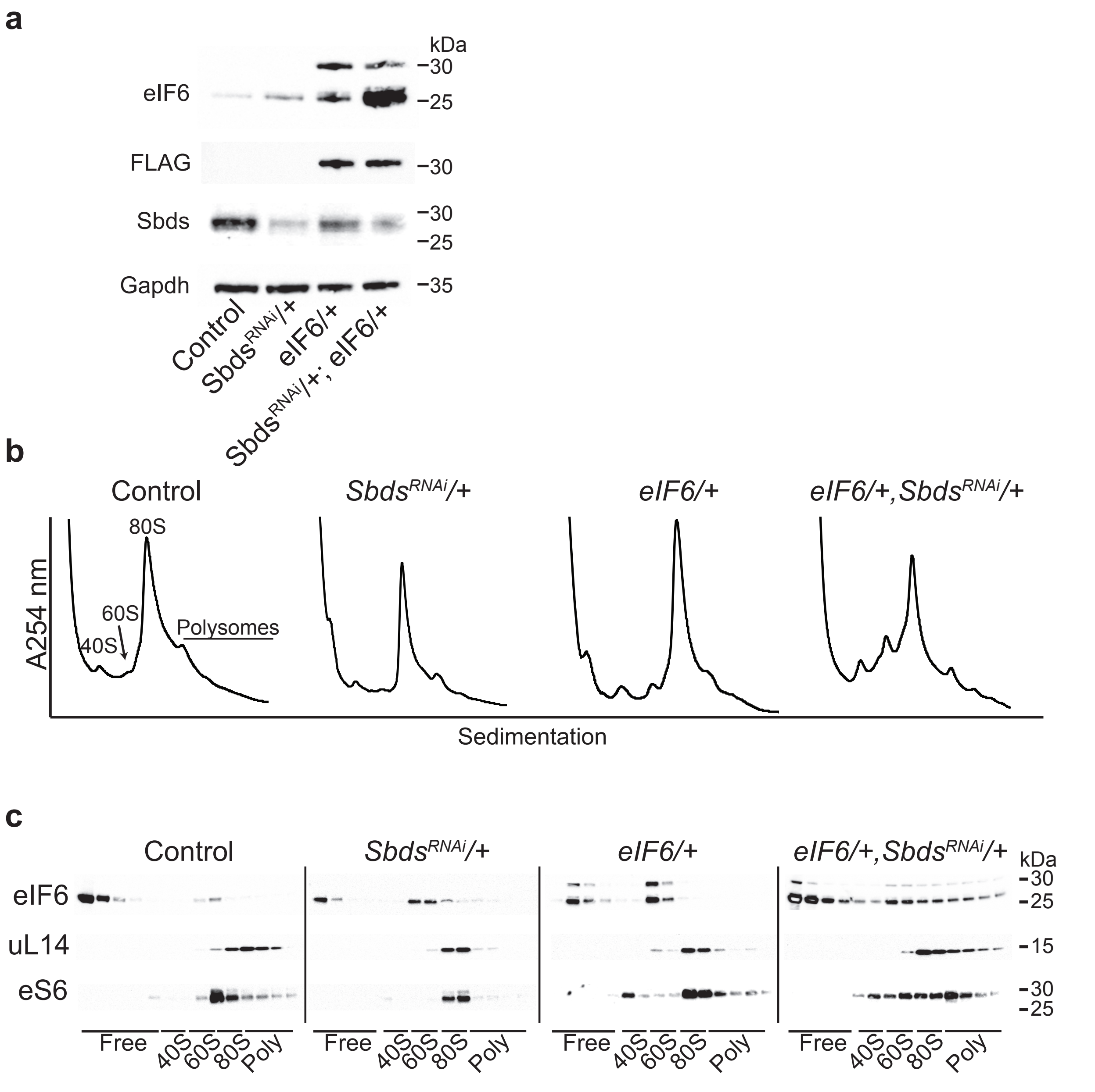

Supplementary Figure 3

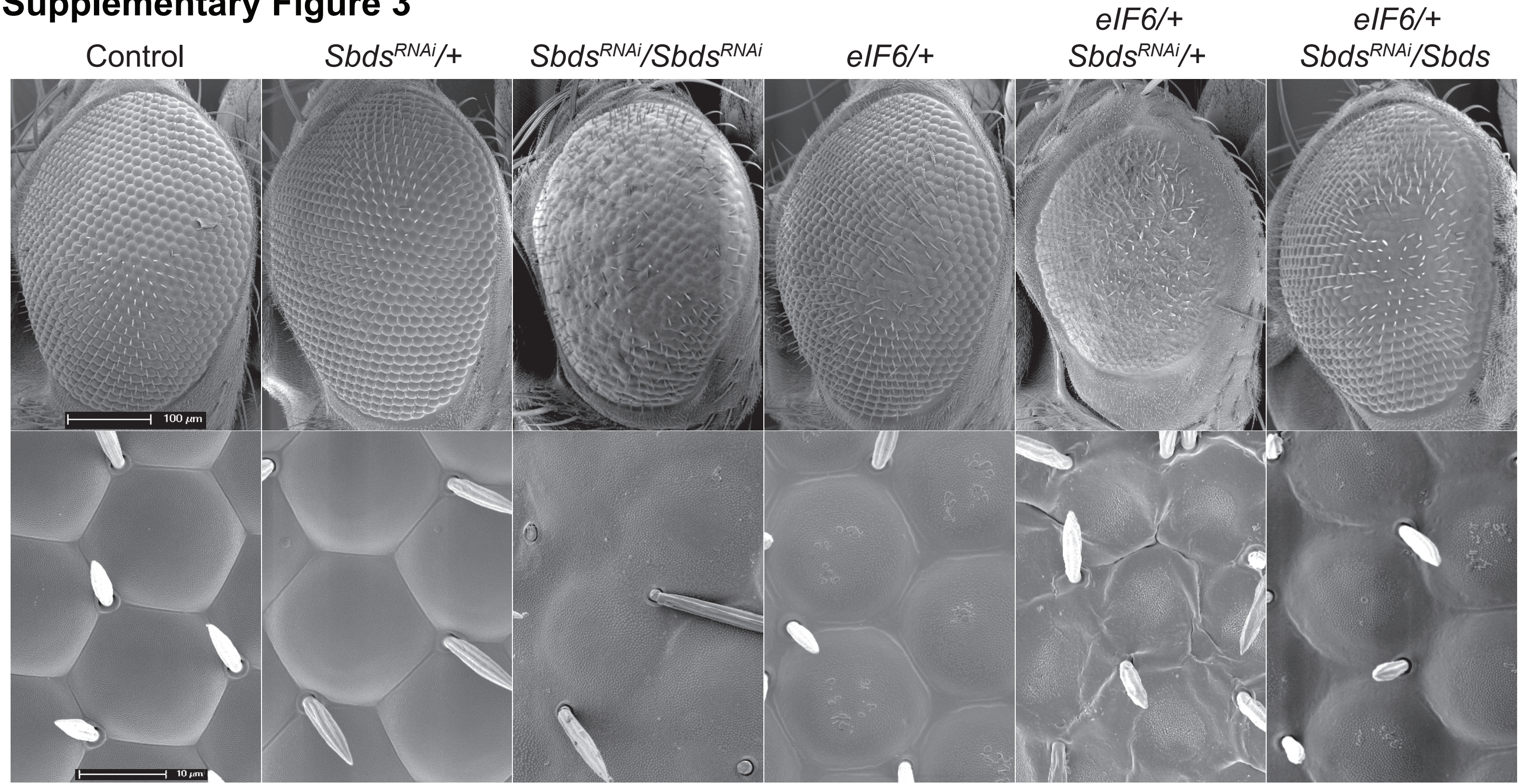

Supplementary Figure 4

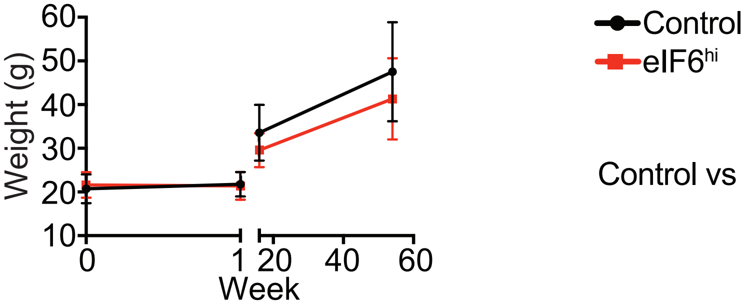

	1w	16w	54w
Control vs eIF6^{hi}	*ns*	*ns*	*P*=0.02598

Supplementary Figure 5

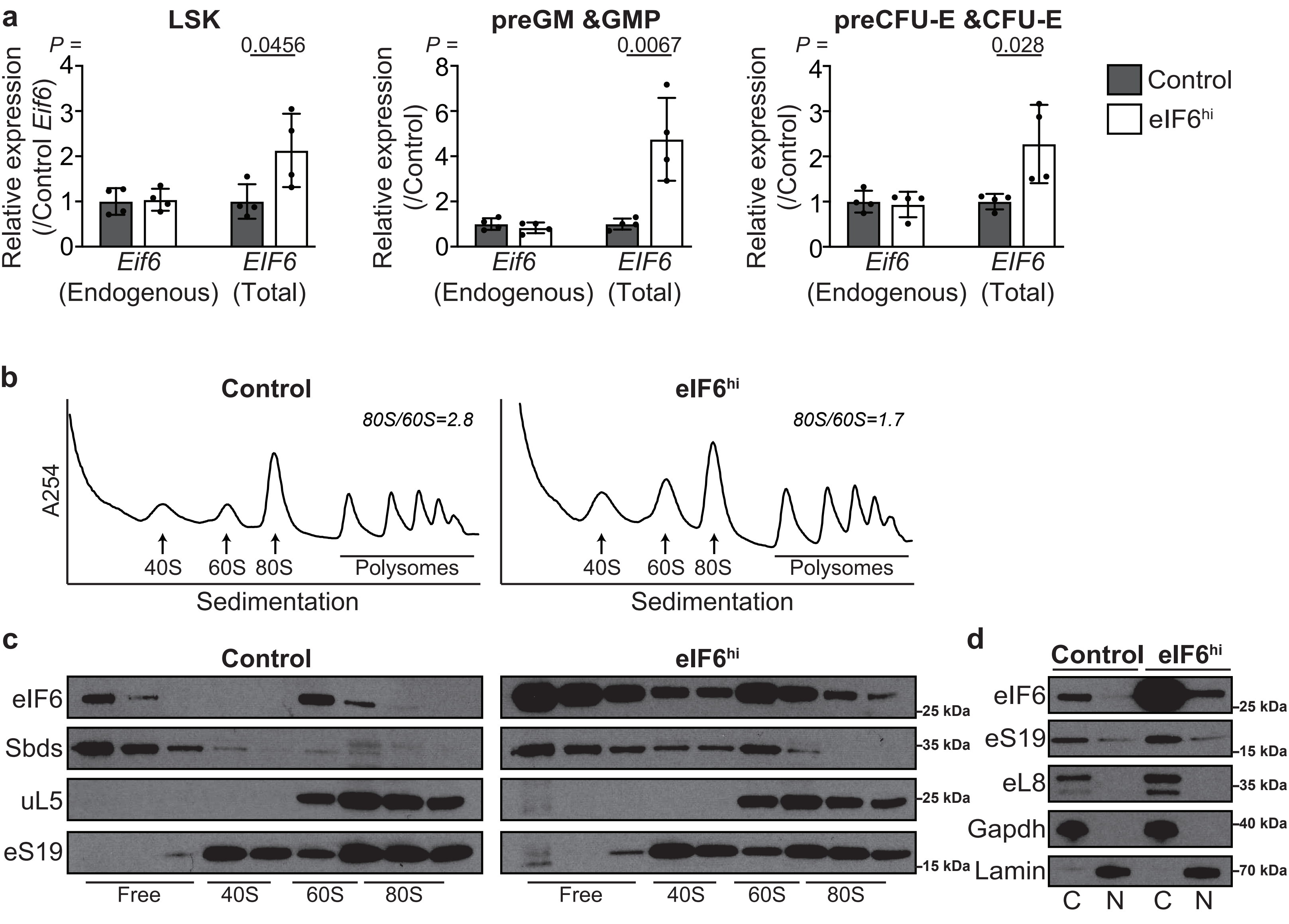

Supplementary Figure 6

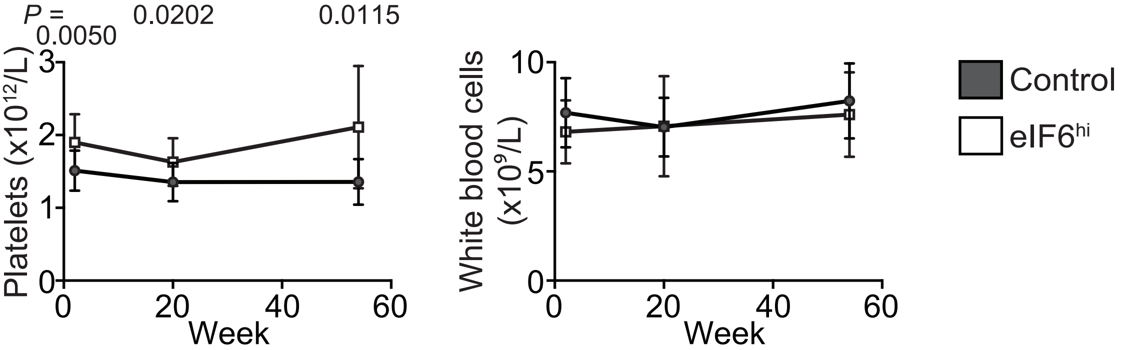

Supplementary Figure 7

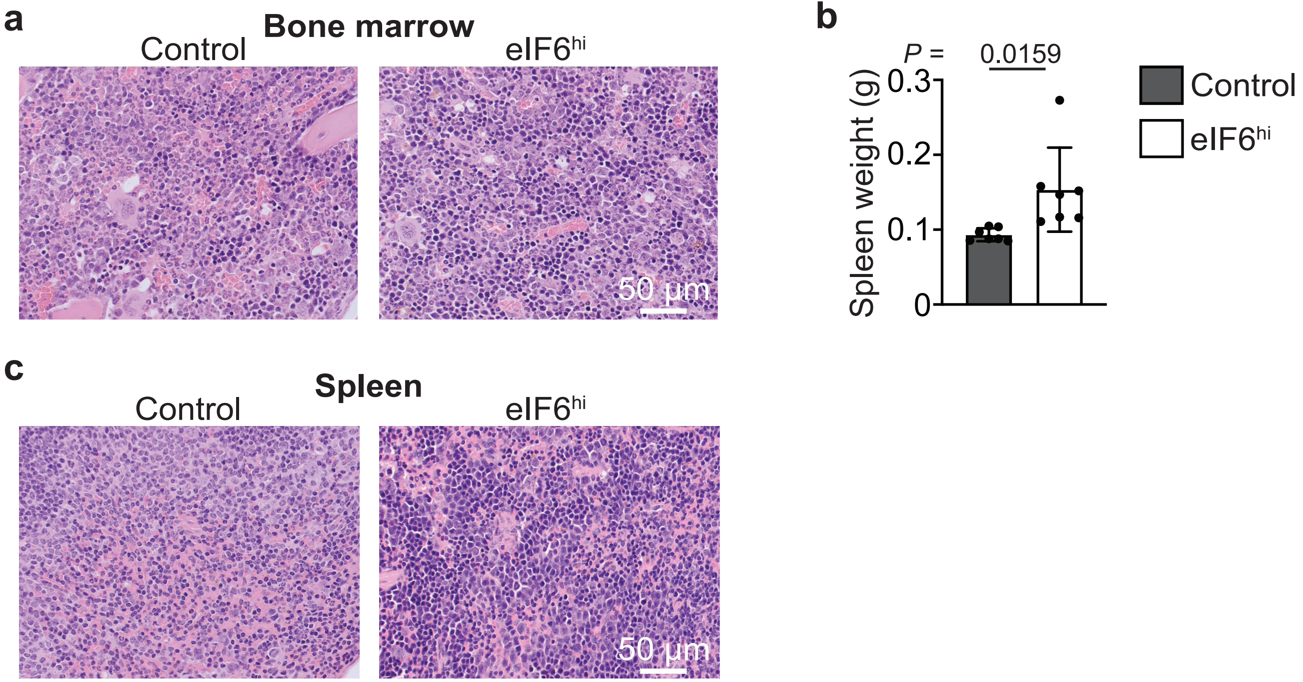

Supplementary Figure 8

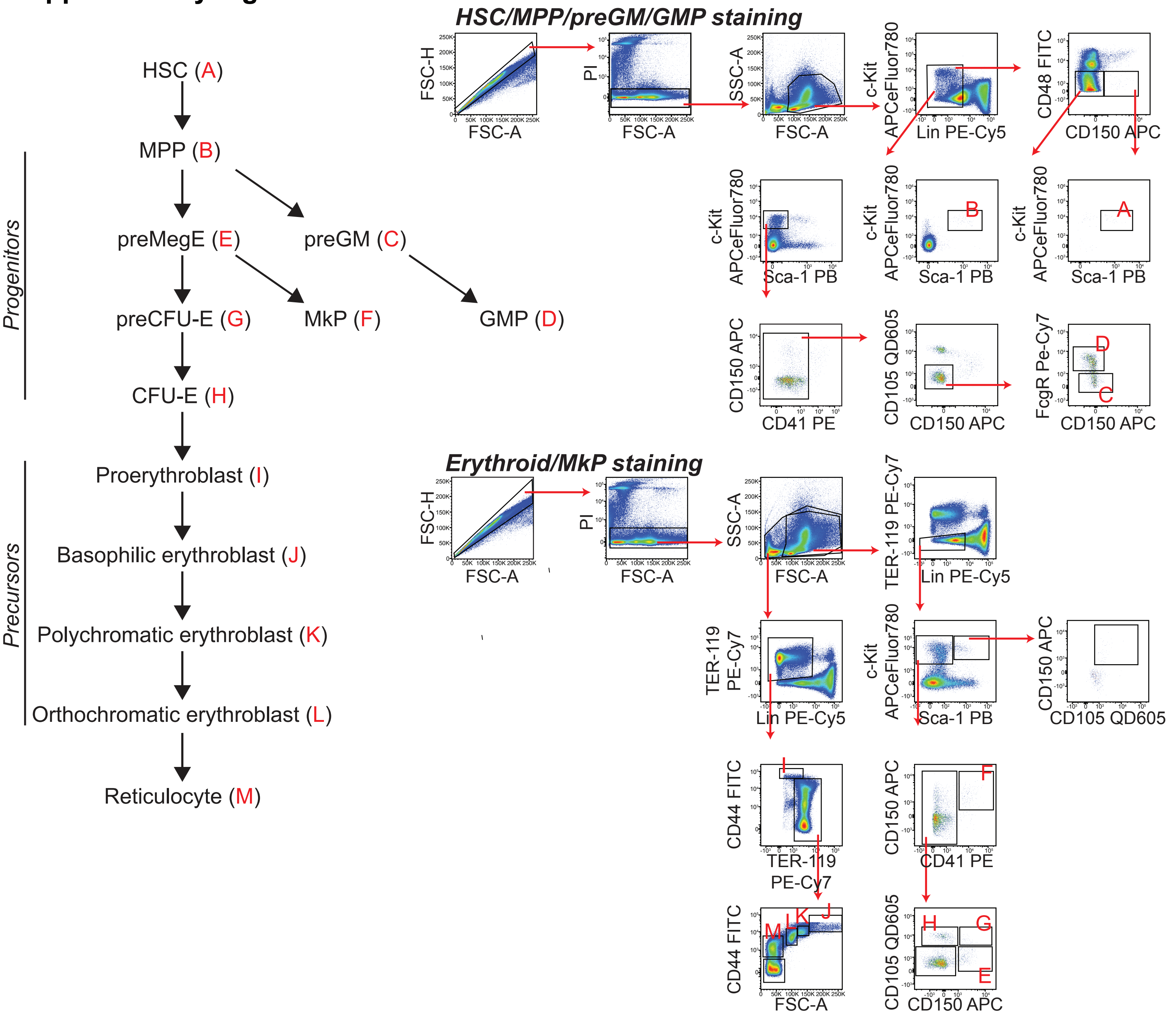

Supplementary Figure 9

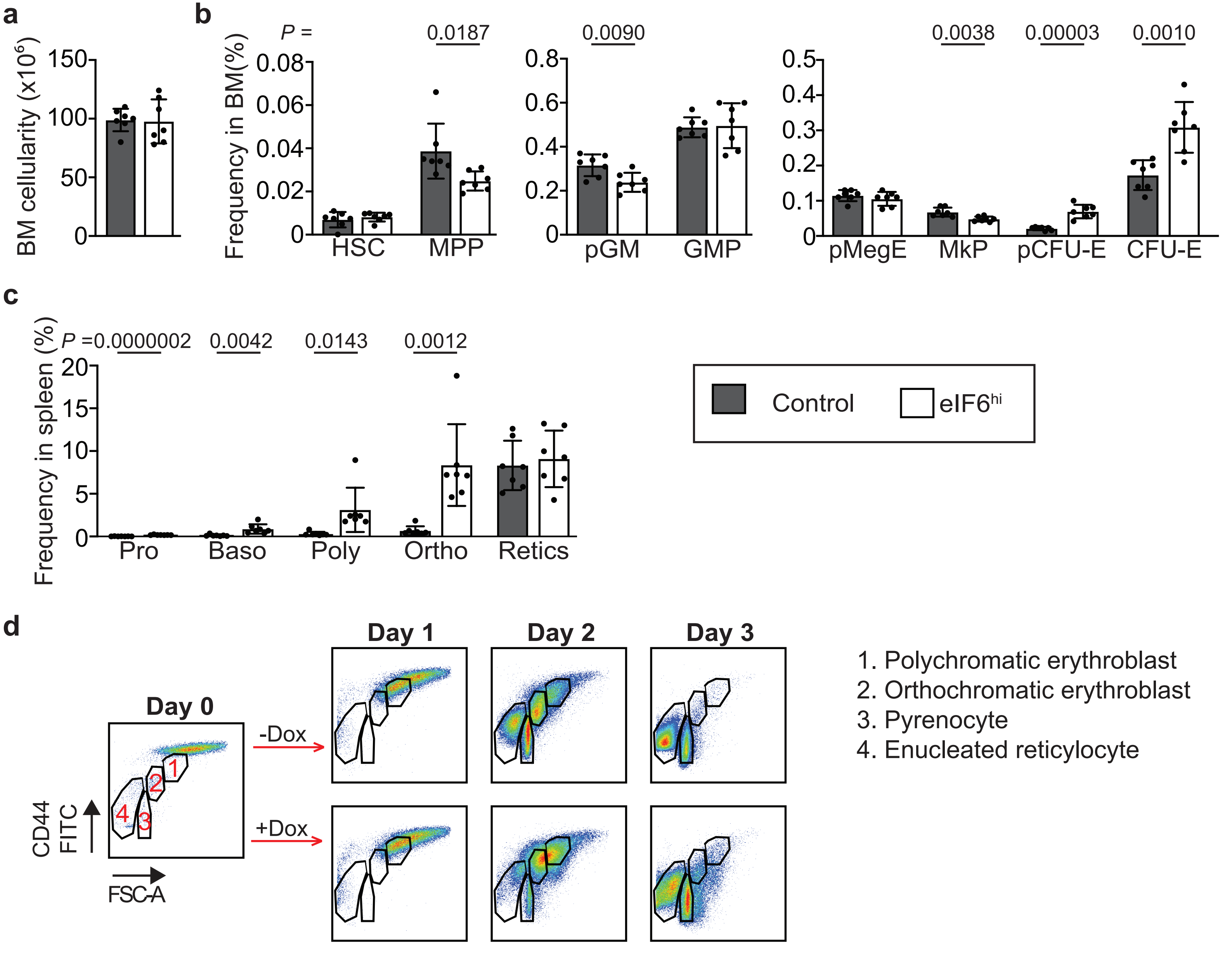

Supplementary Figure 10

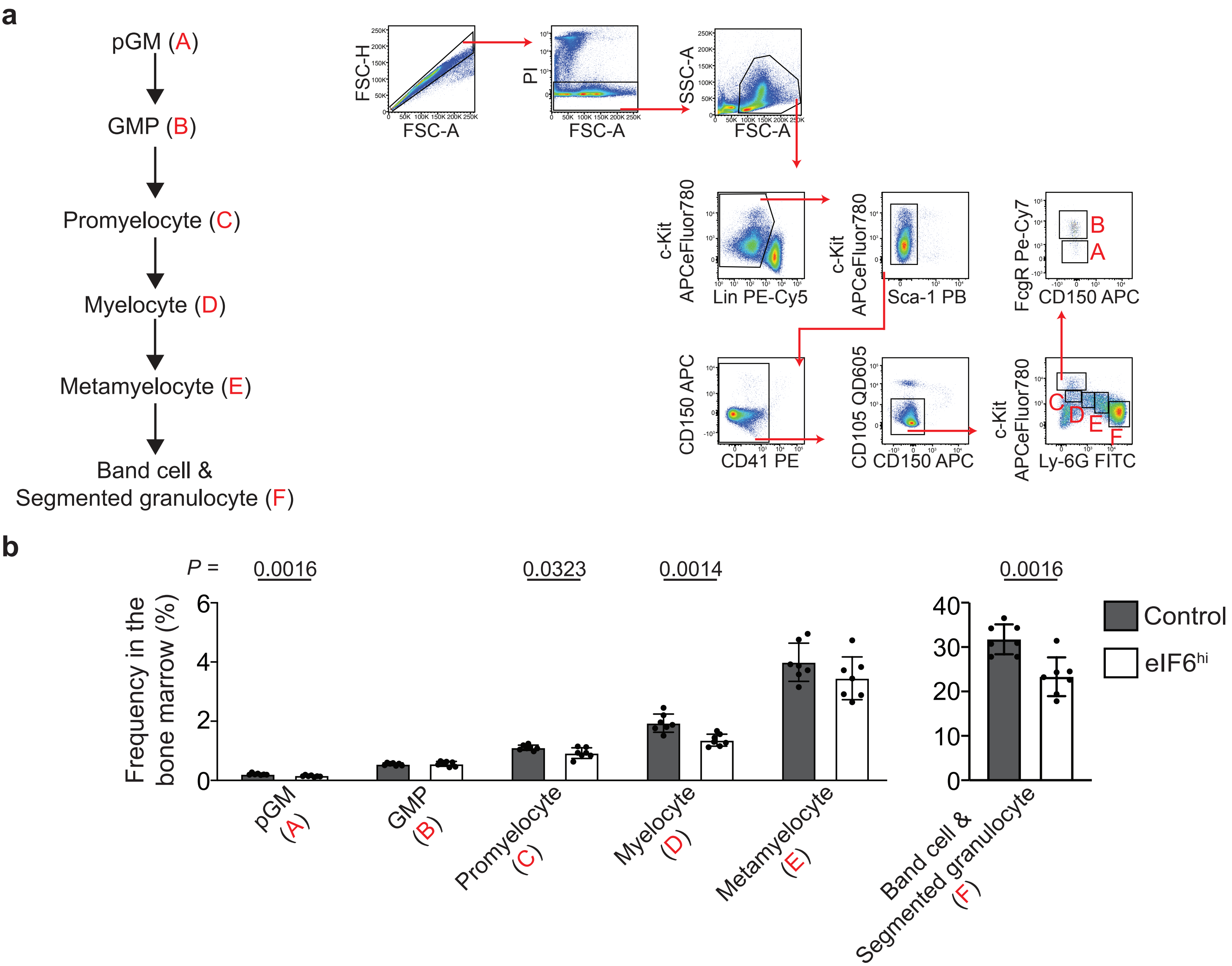

Supplementary Figure 11

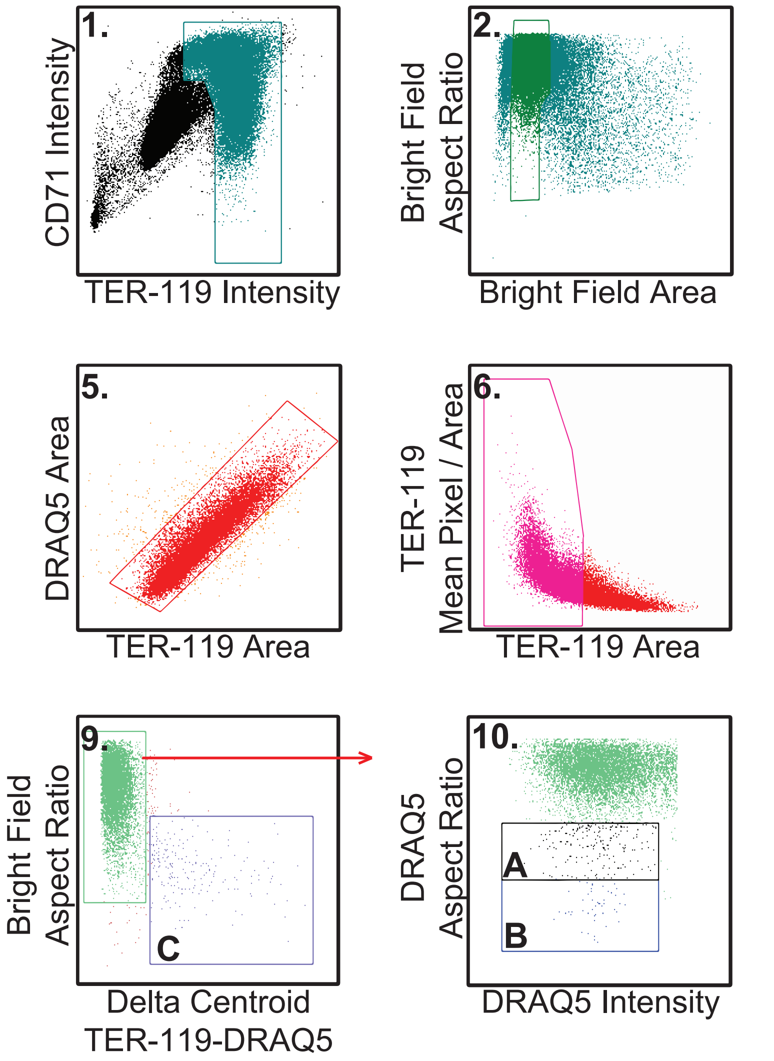

Orthochromatic erythroblast enucleation

A. Early phase

B. Intermediate phase

C. Late phase

Supplementary Figure 12

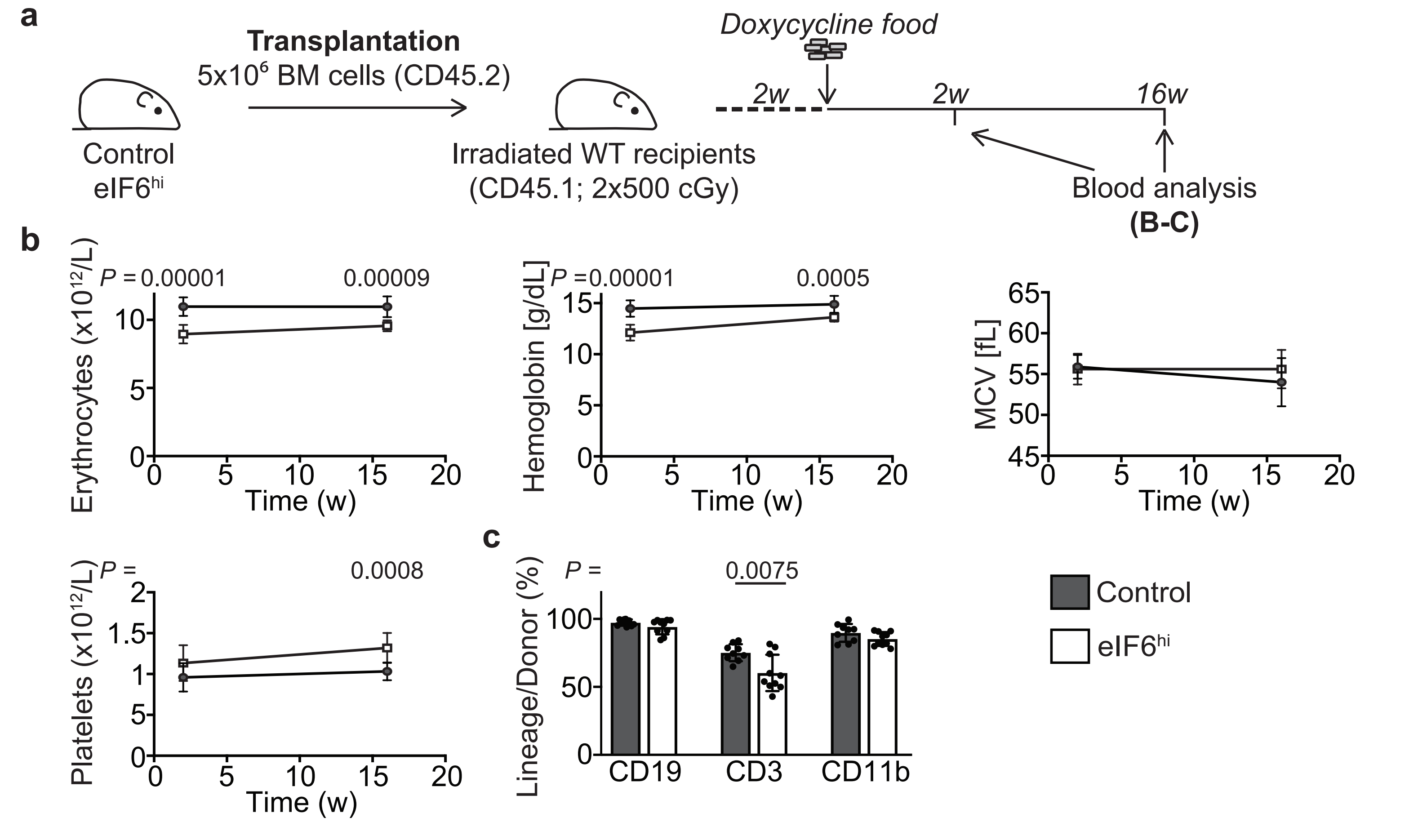

Supplement: Supplementary file 1 — Supplementary Information [file 41467_2022_29214_MOESM1_ESM.pdf]
