# Supplementary material for: eIF6 rebinding dynamically couples ribosome maturation and translation
Source: Nat Commun. 2022 Mar 23;13:1562. doi: 10.1038/s41467-022-29214-7 (PMC8943182; doi:10.1038/s41467-022-29214-7)
Supplement: Supplementary file 2 — Reporting Summary [file 41467_2022_29214_MOESM2_ESM.pdf]

nature portfolio

Corresponding author(s): Alan J. Warren

Last updated by author(s): Mar 1, 2022

Reporting Summary

Nature Portfolio wishes to improve the reproducibility of the work that we publish. This form provides structure for consistency and transparency in reporting. For further information on Nature Portfolio policies, see our Editorial Policies and the Editorial Policy Checklist.

Statistics

For all statistical analyses, confirm that the following items are present in the figure legend, table legend, main text, or Methods section.

n/a	Confirmed	
☐	☒	The exact sample size (*n*) for each experimental group/condition, given as a discrete number and unit of measurement
☐	☒	A statement on whether measurements were taken from distinct samples or whether the same sample was measured repeatedly
☐	☒	The statistical test(s) used AND whether they are one- or two-sided<br>*Only common tests should be described solely by name; describe more complex techniques in the Methods section.*
☒	☐	A description of all covariates tested
☒	☐	A description of any assumptions or corrections, such as tests of normality and adjustment for multiple comparisons
☐	☒	A full description of the statistical parameters including central tendency (e.g. means) or other basic estimates (e.g. regression coefficient) AND variation (e.g. standard deviation) or associated estimates of uncertainty (e.g. confidence intervals)
☐	☒	For null hypothesis testing, the test statistic (e.g. *F*, *t*, *r*) with confidence intervals, effect sizes, degrees of freedom and *P* value noted<br>*Give P values as exact values whenever suitable.*
☒	☐	For Bayesian analysis, information on the choice of priors and Markov chain Monte Carlo settings
☒	☐	For hierarchical and complex designs, identification of the appropriate level for tests and full reporting of outcomes
☒	☐	Estimates of effect sizes (e.g. Cohen's *d*, Pearson's *r*), indicating how they were calculated

Our web collection on statistics for biologists contains articles on many of the points above.

Software and code

Policy information about availability of computer code

Data collection	CryoEM data was collected using EPU software (Thermo Fisher v1.11.0)<br>Flow cytometry data was collected using FACSDiva software (BD)
Data analysis	RELION v3.1, MotionCor2v1.0.5, CTFFIND4.1, ChimeraX v1.0, FlowJo v10.1r7 (Tree Star), IDEAS v6.2 (Merck), ImageJ 1.49v and 2.1.0v, Photoshop CS5 (Adobe), Illustrator CS6 (Adobe), PRISM 8 and 9 (GraphPad).

For manuscripts utilizing custom algorithms or software that are central to the research but not yet described in published literature, software must be made available to editors and reviewers. We strongly encourage code deposition in a community repository (e.g. GitHub). See the Nature Portfolio guidelines for submitting code & software for further information.

Data

Policy information about availability of data

All manuscripts must include a data availability statement. This statement should provide the following information, where applicable:

- Accession codes, unique identifiers, or web links for publicly available datasets
- A description of any restrictions on data availability
- For clinical datasets or third party data, please ensure that the statement adheres to our policy

The authors confirm that all relevant data is included in the manuscript or supplementary information. Source data are provided with this paper.

Field-specific reporting

Please select the one below that is the best fit for your research. If you are not sure, read the appropriate sections before making your selection.

- [x] Life sciences
- [ ] Behavioural & social sciences
- [ ] Ecological, evolutionary & environmental sciences

For a reference copy of the document with all sections, see nature.com/documents/nr-reporting-summary-flat.pdf

Life sciences study design

All studies must disclose on these points even when the disclosure is negative.

Sample size	No statistical methods were used to pre-determine sample size. The sample sizes were instead based on extensive experience with similar experiments in our laboratory (PMID: 31151987, PMID: 25987256, PMID: 31115337)
Data exclusions	No data were excluded from the analysis.
Replication	Reported results were consistently replicated across multiple replicate experiments. Key experiments were performed independently at least twice. The exact number of experimental animals analyzed can be found in the Figure legends.
Randomization	Flies of the same genotype were randomly allocated to experimental group. Randomization was not applied to the animal studies; genotyped sex and age-matched littermate animals were used for control and experimental arms. Cells for peripheral blood and flow cytometry analysis were harvested from individual mice in a randomized order.
Blinding	Experiments were not performed blinded to ensure equal presentation of different genotypes in experimental groups.

Reporting for specific materials, systems and methods

We require information from authors about some types of materials, experimental systems and methods used in many studies. Here, indicate whether each material, system or method listed is relevant to your study. If you are not sure if a list item applies to your research, read the appropriate section before selecting a response.

Materials & experimental systems

n/a	Involved in the study	
[ ]	[x]	Antibodies
[x]	[ ]	Eukaryotic cell lines
[x]	[ ]	Palaeontology and archaeology
[ ]	[x]	Animals and other organisms
[x]	[ ]	Human research participants
[x]	[ ]	Clinical data
[x]	[ ]	Dual use research of concern

Methods

n/a	Involved in the study	
[x]	[ ]	ChIP-seq
[ ]	[x]	Flow cytometry
[x]	[ ]	MRI-based neuroimaging

Antibodies

Antibodies used	Listed in detail in Supplementary Tables 1 and 2.
Validation	The Drosophila Sbds antibody is verified in Supplementary Figure 2. Other antibodies are commercially available and validated by the manufacturer.<br>Myeloid precursor, erythroid precursor and hematopoietic stem and progenitor cell stainings using these antibodies have been described previously: PMID: 23024276. PMID: 19805084. PMID: 18371379.<br>Immunoblotting using these antibodies has been described previously: PMID: 34413298. PMID: 31151987. PMID: 25987256.

Animals and other organisms

Policy information about studies involving animals; ARRIVE guidelines recommended for reporting animal research

Laboratory animals	This study reports the generation of a novel transgenic mouse strain (see Material and Methods section for further details). These mice were generated at the Wellcome Trust Sanger Institute (Hinxton), and the experimental mice were backcrossed into C57BL/6 background for at least three generations. Transplantation experiments were performed using B6.SJL-Ptprca (CD45.1) mice as hosts. All experiments were performed using adult (8-12 weeks old) female and male mice with littermate controls. Mice were maintained in a standard SPF facility (12 light/12 dark cycle, 19-23 degrees Celsius with 40-60 % humidity)<br><br>Drosophila strains are listed in Supplementary Table 4. Either adult flies or larvae were used as described in the Material and Methods section and Figure legends. Studies were performed on randomly chosen female and male flies. Eye photos in Fig 3B and Fig

	S3 were obtained using female flies but a similar phenotype was observed in males.
Wild animals	No wild-type animals were used in this study.
Field-collected samples	No field-collected samples were used in this study.
Ethics oversight	Animal procedures were regulated under UK Home Office Animals (Scientific Procedures) Act 1986 under project license 70/8406.

Note that full information on the approval of the study protocol must also be provided in the manuscript.

Flow Cytometry

Plots

Confirm that:

- [x] The axis labels state the marker and fluorochrome used (e.g. CD4-FITC).
- [x] The axis scales are clearly visible. Include numbers along axes only for bottom left plot of group (a 'group' is an analysis of identical markers).
- [x] All plots are contour plots with outliers or pseudocolor plots.
- [x] A numerical value for number of cells or percentage (with statistics) is provided.

Methodology

Sample preparation	We isolated bone marrow cells by crushing hips, femurs and tibias in PBS (Thermo Fisher Scientific) supplemented with fetal calf serum (FCS; 2 % Thermo Fisher Scientific) and EDTA (2 mM; Thermo Fisher Scientific). Isolated cells were filtered through a 70 µm cell strainer (Thermo Fisher Scientific). Antibody labeling was performed in PBS (+2 % FCS) for 30 min on ice. Thiazole orange (5 µM; Biotium) was included during antibody staining where specified. Antibodies are listed in the Supplementary Table 1. Erythrocytes were removed from peripheral blood by Dextran sedimentation (2 % in PBS; Merck) and ACK lysis buffer (Thermo Fisher Scientific) before antibody labeling.
Instrument	Experiments were performed using FACSARIA III cell sorter (BD Biosciences) and LSRFortessa flow cytometer (BD Biosciences)
Software	Experiments were analysed using FlowJo software (Tree Star, v10.1r7).
Cell population abundance	Cell population abundances are reported in detail in Figures and Supplementary Figures, and are in line with published data by our lab and others. Purity of sorted cells was verified by post-sort flow cytometry analysis and by cytospins/microscopy and in vitro cell differentiation assays where applicable.
Gating strategy	The exact gating strategies are shown in the respective Figures or alternatively in Supplementary Figures 8 and 10.<br><br>Cells were identified by FSC/SSC gating to exclude debris and cell aggregates. Dead cells were excluded using propidium iodide. The gate corresponding to lineage-negative cells was determined using either c-Kit+ or TER-119+ cells as an internal control.<br>Erythroid precursors were gated as previously described: PMID: 19805084.<br>Myeloid precursors were gated as previously described: PMID: 23024276.<br>Hematopoietic stem and progenitor cells were gated as previously described: PMID: 18371379.

- [x] Tick this box to confirm that a figure exemplifying the gating strategy is provided in the Supplementary Information.
